# Preclinical serum alterations and tissue changes in protein and gene expression of early cerebrospinal fluid-validated biomarkers in scrapie

**DOI:** 10.1186/s13567-026-01759-1

**Published:** 2026-06-02

**Authors:** Sonia Pérez-Lázaro, Tomás Barrio, Eloisa Sevilla, Rosa Bolea, Juan J. Badiola

**Affiliations:** 1 Centro de Encefalopatías y Enfermedades Transmisibles Emergentes (CEETE), Facultad de Veterinaria, Universidad de Zaragoza, Miguel Servet 177, 50013 Zaragoza, Spain; 2 Instituto Universitario de Investigación Mixto Agroalimentario de Aragón (IA2) UNIZAR-CITA, Zaragoza, Spain; 3 Instituto de Investigación Sanitaria de Aragón (IIS-Aragón), Zaragoza, Spain; 4 UMR INRAE-ENVT 1225 Interactions Hôtes-Agents Pathogènes, École Nationale Vétérinaire de Toulouse, 31076 Toulouse, France

**Keywords:** Prion, neurodegenerative diseases, scrapie, preclinical diagnosis, serum biomarkers, SYNCRIP, PLD3, CTSD, SPP1, C4

## Abstract

Identifying reliable biomarkers for prion diseases remains a challenge, particularly for early detection in accessible body fluids. A recent mass spectrometry study on the cerebrospinal fluid proteome identified and validated five proteins significantly dysregulated in the preclinical stage of naturally scrapie-affected sheep. In this study, we quantified these proteins in serum and analysed their distribution and gene expression in the central nervous system, correlating these results with characteristic prion-related neuropathological features. Significantly elevated serum levels of synaptotagmin binding, cytoplasmic RNA interacting protein (SYNCRIP), phospholipase D3 (PLD3) and cathepsin D (CTSD) were detected in preclinical animals, mirroring previous cerebrospinal fluid findings. Central nervous system analyses on these three proteins together with osteopontin (SPP1) and complement component 4 (C4) revealed early and region-specific reduced immunoreactivity alongside upregulated gene expression in scrapie-affected animals, correlating significantly with prion-associated neuropathological features. Together, these findings highlight the potential of SYNCRIP, PLD3 and CTSD as promising minimally invasive biomarkers to diagnose prion diseases from the preclinical stage and provide new insights into the spatiotemporal regulation of the five proteins in the central nervous system throughout the progression of disease. Further research is needed to clarify the peripheral biomarker dynamics in relation to the neurodegenerative pathology.

**Supplementary Information:**

The online version contains supplementary material available at 10.1186/s13567-026-01759-1.

## Introduction

Prion diseases or transmissible spongiform encephalopathies (TSE) are fatal neurodegenerative disorders characterised by the misfolding of the physiological cellular prion protein (PrP^C^) into its pathogenic isoform (PrP^Sc^). This structural conversion leads to the accumulation of PrP^Sc^, primarily in the central nervous system (CNS), triggering spongiform neurodegeneration, neuronal loss and gliosis [1]. TSE affect a wide variety of species, including Creutzfeldt–Jakob disease and its sporadic form (sCJD) in humans, bovine spongiform encephalopathy (BSE) in cattle and scrapie in small ruminants. Among these, scrapie serves as the prototype of prion diseases and has been extensively researched and used as a model of these disorders. One key feature of scrapie is the early deposition of PrP^Sc^ in peripheral lymphoid tissues, allowing the detection of preclinical infected asymptomatic animals through rectal mucosa biopsies [2]. Beyond TSE, several other neurodegenerative disorders, such as Alzheimer’s disease (AD), Parkinson’s disease (PD) and amyotrophic lateral sclerosis (ALS), share common misfolding and aggregation mechanisms of specific proteins: beta-amyloid and tau in AD, alpha-synuclein in PD, and SOD1 and TDP-43 in ALS [3–5].

Recent advances in ultrasensitive detection methods, such as protein misfolding cyclic amplification (PMCA) and real-time quaking-induced conversion (RT-QuIC), have significantly improved prion diagnosis by detecting the specific PrP^Sc^ in various biological samples [6]. However, variability in the extent of PrP^Sc^ distribution, as well as differences in host genetics and prion strains, complicates diagnosis based solely on PrP^Sc^ detection [7]. This highlights the importance of identifying additional molecular biomarkers to complement PrP^Sc^ as indicators of disease before the onset of clinical signs and as trackers of disease progression over time. For this purpose, the cerebrospinal fluid (CSF), being in direct contact with the CNS and reflecting its biochemical changes, represents a promising source for studying biomarkers of prion pathogenesis. However, existing surrogate biomarkers of neuronal damage in human CSF, such as 14–3-3 and tau proteins, lack specificity and are primarily detectable in later disease stages [8]. Therefore, identifying novel biomarkers capable of detecting prion disease before the onset of clinical signs and of tracking disease progression, particularly in more accessible body fluids such as blood, is crucial.

Recently, we conducted a mass spectrometry analysis of the CSF proteome of scrapie-affected sheep, comparing both preclinical and clinical stages of disease versus healthy controls [9]. Focussing specifically on the preclinical stage in which sheep exhibited no clinical signs, we identified numerous significantly dysregulated proteins in these preclinical animals when compared with healthy ones. Additionally, several metabolic pathways were altered in that stage, including oxidative stress response, ion transport and inflammatory processes, suggesting early molecular changes before clinical onset. Among all dysregulated proteins, on the basis of fold change and significance values and previously reported data on prion and other human neurodegenerative diseases, five proteins were selected for further investigation: synaptotagmin binding, cytoplasmic RNA interacting protein (SYNCRIP), phospholipase D family member 3 (PLD3), cathepsin D (CTSD), osteopontin (SPP1) and complement component 4 (C4). These proteins were subsequently validated by enzyme-linked immunosorbent assay (ELISA) in the CSF. Interestingly, all five proteins were found to be upregulated in the CSF of preclinical sheep compared with healthy controls, maintaining elevated levels in the clinical stage of the disease, except for CTSD, whose concentration returned to the levels observed in healthy sheep. These results highlighted their potential as early diagnostic biomarkers for prion diseases and potentially other neurodegenerative disorders. However, the behaviour of these potential biomarkers in other body fluids, particularly those that are more accessible and less invasive to obtain than CSF, and the underlying mechanisms driving their upregulation in the CSF remained unexplored.

To this end, in the present study, we first analysed the concentration of the five previously CSF-validated proteins (SYNCRIP, PLD3, CTSD, SPP1 and C4) in the serum of preclinical and clinical scrapie-affected sheep, alongside healthy controls, using ELISA. This analysis aimed to assess whether these proteins also reflect disease-associated changes in a peripheral, easily accessible body fluid, and to evaluate their potential as less-invasive early diagnostic biomarkers of disease. Moreover, we further investigated the potential role of these proteins in the neuropathology of prion diseases, by exploring their protein expression in the CNS of preclinical and clinical naturally scrapie-affected sheep, comparing the data with that of healthy animals. Specifically, an immunohistochemical study was performed to analyse protein deposition and distribution throughout several regions of the CNS, correlating the results with neuropathological features of prion diseases. To complement this, the expression levels of the genes encoding the proteins of interest were also assessed by real-time quantitative PCR (RT-qPCR) for an in-depth evaluation of whether gene expression changes reflected the protein expression levels in the most scrapie-affected CNS regions.

## Materials and methods

### Animals

Female sheep from the Spanish Rasa Aragonesa breed, ranging from 3 to 6 years and displaying the ARQ/ARQ *PRNP* genotype, were included in the present research study. Scrapie-affected sheep were selected from naturally scrapie-affected flocks. Preclinical sheep with no clinical signs of disease were detected by PrP^Sc^ immunohistochemical analysis of rectal biopsies, owing to its early deposition in sheep lymphoid tissue [10]. Typical scrapie clinical signs such as pruritus and ataxia were identified in clinical sheep, and the infection was confirmed by immunohistochemical detection of PrP^Sc^ in the brain, as detailed below. Healthy sheep were sampled from flocks with no history of scrapie outbreaks, matching the age and genotype of the scrapie-affected sheep. Specifically, for the protein detection analysis in serum and gene expression analyses in the CNS, 21 animals were used: 8 healthy sheep as controls, 5 sheep in the preclinical stage of scrapie and 8 sheep in the clinical stage showing notable signs of scrapie. For the histopathological and immunohistochemical analyses, 19 animals were selected: 7 healthy controls, 5 sheep in the preclinical stage and 7 clinical sheep.

### Sample collection

Blood samples were collected through jugular venipuncture using vacutainer tubes with no anticoagulant. They were processed immediately after and centrifuged at 3100 rpm for 10 min, and the sera were aliquoted for preservation at −80 °C until use. Then, animals were sacrificed by intravenous injection of sodium pentobarbital and exsanguination. During the necropsy, samples from six areas of the CNS were taken: cervical spinal cord, medulla oblongata at the level of the obex, cerebellum, thalamus, hippocampus and frontal cortex. These were divided in half, given that the lesion pattern of scrapie is bilateral [11]; one half was immersed in formaldehyde (10%) for fixation and subsequent histopathological and immunohistochemical analyses, and the other half of CNS samples was divided once more: a portion of the tissue was stabilised in RNAlater solution (Thermo Fisher Scientific, USA) and then stored at −80 °C for RNA extraction, and the rest was directly frozen at −80 °C for protein analysis.

### ELISA

For serum protein analysis, the same ELISA kits used for CSF validation were used here, following the manufacturer’s protocol for serum samples and details already described [9]. Kit references for each protein are as follows, including the serum dilution (sample volume: pH 7 phosphate buffered saline volume): SYNCRIP (1:4, #E14H0387, BlueGene, China), PLD3 (Undiluted, #MBS9361401, MyBioSource, USA), CTSD (1:4, #E14C0651, BlueGene, China), SPP1 (1:1, #E14O0005, BlueGene, China) and C4 (1:1, #E14C0012, BlueGene, China). All samples were analysed in triplicate.

### Histopathological and immunohistochemical analyses

Formalin-fixed CNS tissue from each region and sheep was cut into thin portions and embedded in paraffin. Then, paraffin blocks were sliced into 4-µm-thick sections and kept drying at 37 °C for 24 h. Haematoxylin–eosin (HE) staining was performed for neuropathological changes and spongiosis evaluation. Immunohistochemistry (IHC) for PrP^Sc^ detection was accomplished using the primary monoclonal antibody L42 (1/500, R-Biopharm, Germany), which recognises amino acid residues 145–163 of the ovine PrP sequence, as previously described [12].

For the IHC analysis of the five proteins studied, a heat-mediated antigen retrieval was performed in a PT-Link equipment (Dako Agilent, USA), for 20 min at 96 °C in citrate buffer (Dako Agilent, USA) for all proteins except for C4, in which case a Tris–ethylenediaminetetraacetic acid (EDTA) buffer (Dako Agilent, USA) for 15 min was needed. The references of the primary antibodies are listed as follows, indicating the dilution used in EnVision FLEX Antibody diluent (Dako Agilent, USA): SYNCRIP (1/100, monoclonal, #ab184946, Abcam, USA), PLD3 (1/200, polyclonal, #DF9753, Affinity Biosciences, USA), cathepsin D (1/200, polyclonal, #bs-1615R, Bioss Antibodies, USA), SPP1 (1/100, polyclonal, #AF0227, Affinity Biosciences, USA) and C4 (1/150, polyclonal, #NBP2-14,893, Novus Biologicals, USA). Slices were incubated with the primary antibody solution at 4 °C overnight, and an anti-rabbit enzyme-conjugated secondary antibody (EnVision + System-HRP Labelled Polymer Anti-rabbit, Dako Agilent, USA) was added for 30 min at room temperature (RT), using then diaminobenzidine (DAB; Liquid DAB + Substrate Chromogen System, Dako Agilent, USA) for 10 min as the chromogen. Slices were counterstained for 5 min with haematoxylin, dehydrated and mounted with DPX mounting media (Dako Agilent, USA). To control for non-specific binding, appropriate negative controls were included in all IHC assays by substituting the primary antibody with a species-matched IgG isotype control (#026102, Thermo Fisher Scientific, USA), at the same concentration and following identical protocols. No immunolabelling was observed under these conditions (Additional file 1).

Slices were also immunoassayed for glial fibrillary acidic protein (GFAP; 1/500, polyclonal, Dako Agilent, USA), as an astrocyte marker, and for ionised calcium binding adaptor molecule 1 (Iba1; 1/1000, polyclonal, Wako, USA), as a microglia marker, following already described protocols [13].

Histopathological and immunohistochemical evaluations were conducted in six brain regions, and several representative areas per region were evaluated: cervical spinal cord, including white and grey matters; medulla oblongata at the level of the obex, including the cuneate nucleus, dorsal motor nucleus of vagus and inferior olive nucleus; cerebellum, comprising the molecular layer, Purkinje cell layer, granular layer and white matter; thalamus, covering the ventral, central and dorsal areas; hippocampus, including the dentate gyrus, cornu ammonis (CA) 4 to 1 and stratum lacunosum-moleculare (SLM); and frontal cortex, including white and grey matters. Neuropathological markers (PrP^Sc^ deposits, spongiosis, astrogliosis and microgliosis) and the expression of the selected proteins were evaluated using a semiquantitative scoring system. Staining intensity and distribution were blindly assessed using a Zeiss Axioskop 40 optical microscope (Zeiss, Germany), and scores ranging from 0 (absence of staining or vacuolisation) to 5 (very intense and widespread staining or vacuolisation) were assigned, as previously described [14, 15]. For each parameter, scores were averaged per region or subarea and graphically represented as mean ± standard deviation.

### Western blot

Western blot (WB) analyses were performed to qualitatively assess antibody reactivity against the studied proteins prior to their IHC application. Analyses were conducted on frozen thalamic tissue, as this region is one of the most consistently affected by scrapie pathology [16]. Tissue was homogenised at 10% (w/v) in lysis buffer with protease inhibitor (Roche, Switzerland), using TeSeE grinding tubes and a TeSeEPrecess 48 homogeniser (BioRad, USA). Protein concentration of each sample was calculated using the PierceTM BCA Protein Assay (Thermo Fisher Scientific, USA), following the manufacturer’s protocol. Then, 30 µg of total protein were mixed with a 2 × Laemmli Sample buffer (Bio-Rad, USA), heated at 95 °C for 5 min and loaded into a Criterion XT Precast 12% Bis–Tris gel (Bio-Rad, USA). The electrophoresis was set to 80 V for 30 min and uploaded to 120 V for 90 min or until the dye front arrived at the bottom of the gel. In case of complement C4, samples were loaded into a Criterion TGX Precast 7.5% gel (Bio-Rad, USA) and submitted to 80 V for 30 min and 140 V for 70 min. Proteins were transferred to polyvinylidene difluoride (PVDF) membranes (Bio-Rad, USA) using the Trans-Blot Turbo Transfer system (Bio-Rad, USA). The membranes were blocked for 1 h with 2% non-fat dry milk in Tris-buffered saline with 0.1% Tween 20 (TBST) and incubated with the primary antibodies used for IHC at 4 °C overnight. Dilutions in TBST were as follows: SYNCRIP 1/10000, PLD3 1/1500, cathepsin D 1/1000, SPP1 1/1000 and complement C4 1/1000. In the case of PLD3 and SPP1, 0.5% and 1% of non-fat dry milk were added to the primary antibody dilution, respectively. After that, membranes were incubated for 1 h at RT with a peroxidase conjugated anti-rabbit IgG secondary antibody (#31,460, Thermo Fisher Scientific, USA), at a dilution of 1/20000 in TBST. Immunoreactivity signal was developed using the ECL Select Western Blotting detection reagent (Amersham Cytiva, USA) and visualised with the BioSpectrum 815 imaging system (UVP, USA).

### RT-qPCR

Gene expression analyses were performed by RT-qPCR on the two CNS regions most affected by scrapie: medulla oblongata at the level of the obex and thalamus [16]. First, RNAlater preserved tissue from these CNS areas was used for total RNA extraction, using the Direct-zol RNA Miniprep Plus kit (Zymo Research, USA), as previously described [17]. Quality and concentration of RNA were analysed in a NanoDrop spectrophotometer (Thermo Fisher Scientific, USA). RNA purity was confirmed by 260/280 and 260/230 absorbance ratios, with values averaging 1.98 ± 0.06 across all samples. Then, complementary DNA (cDNA) was obtained by retrotranscribing 1 µg of total RNA using the qScript cDNA SuperMix kit (Quantabio, USA), following the manufacturer’s protocol. The genes codifying the five proteins of interest were studied: *SYNCRIP*, *PLD3*, *CTSD*, *SPP1* and *C4*. Additionally, stable genes previously described for their use as housekeeping genes in CNS samples from scrapie-affected sheep were also analysed: *GAPDH* (glyceraldehyde-3-phosphate dehydrogenase), *G6PD* (glucose-6-phosphate dehydrogenase) and *SDHA* (succinate dehydrogenase complex flavoprotein subunit A) [18]. PrimerBLAST [19] and Primer3Plus v3.3.0 [20] were used for primer design. We ensured primers were 100% gene-specific, binding in two contiguous exons separated by a lengthy intron when possible, and melt curves were also analysed for potential primer dimers. Different concentrations of primers were evaluated for better amplification and serial dilutions of cDNA were tested, achieving an efficiency of 90–110%. Primer sequences together with the optimal concentration tested can be found in Additional file 2.

RT-qPCR was performed on the QuantStudio 5 instrument (Thermo Fisher Scientific, USA). Samples were run in triplicate, and the reaction mix was prepared with 15 ng of cDNA, variable primer volume depending on the primer concentration needed (Additional file 2) and 5 µL of RealQ Plus 2 × Master Mix Green, low ROX (Ampliqon, Denmark), following a standard run mode. The QuantStudio Design & Analysis software version 1.5.3 (Thermo Fisher Scientific, USA) was used for cycle threshold (Ct) values calculation. Ct values of potential housekeeping genes were uploaded to the RefFinder platform [21], and the most stable ones were selected (Additional file 3). The mean of *SDHA* and *GAPDH* was calculated for normalisation of obex results, and the mean of *SDHA* and *G6PD* was used for thalamus samples. RT-qPCR data were analysed using the 2^−ΔΔCt^ method [22], as previously applied [17]. Relative expression was calculated using healthy controls as the reference group, and 2^−ΔΔCt^ values were plotted.

### Statistical analyses

Graphical representations and all statistical analyses needed in this study were performed using R (version 4.2.2).

For the ELISA data analysis, intra- and inter-assay coefficients of variation were determined, yielding values below 10% and 15%, respectively. Sample concentrations were calculated on the basis of standard curves fitted with polynomial regression, as previously described [9]. For group comparisons, data normality and homogeneity of variances were assessed using the Shapiro–Wilk and Levene’s tests, respectively. When both assumptions were met, one-way analysis of variance (ANOVA) test was used, followed by Tukey’s honestly significant difference (HSD) test for multiple comparisons. If assumptions were not satisfied, Kruskal–Wallis followed by Dunn’s test was applied. Normally distributed variables are summarised as mean ± standard deviation, whereas non-normally distributed variables are expressed as median ± interquartile range. For IHC analyses, semiquantitative scoring data were specifically analysed using non-parametric statistical methods (Kruskal–Wallis followed by Dunn’s test). To explore potential correlations between protein immunoreactivity levels and neuropathological features, Spearman’s rank correlation coefficients were calculated using the cor function in R, and *p*-values determined with the Hmisc version 5.1.1 R package. Correlation analyses were performed separately for each CNS region, using the semiquantitative IHC scores. R package ggplot2 was used for data visualisation, and all statistical significance levels are denoted in plots as follows: * (*p*-value < 0.05), ** (*p*-value < 0.01), *** (*p*-value < 0.001), and ^ (tendency; *p*-value < 0.1).

## Results

### Serum analysis of potential biomarkers for early prion diagnosis by ELISA

**Figure 1:**
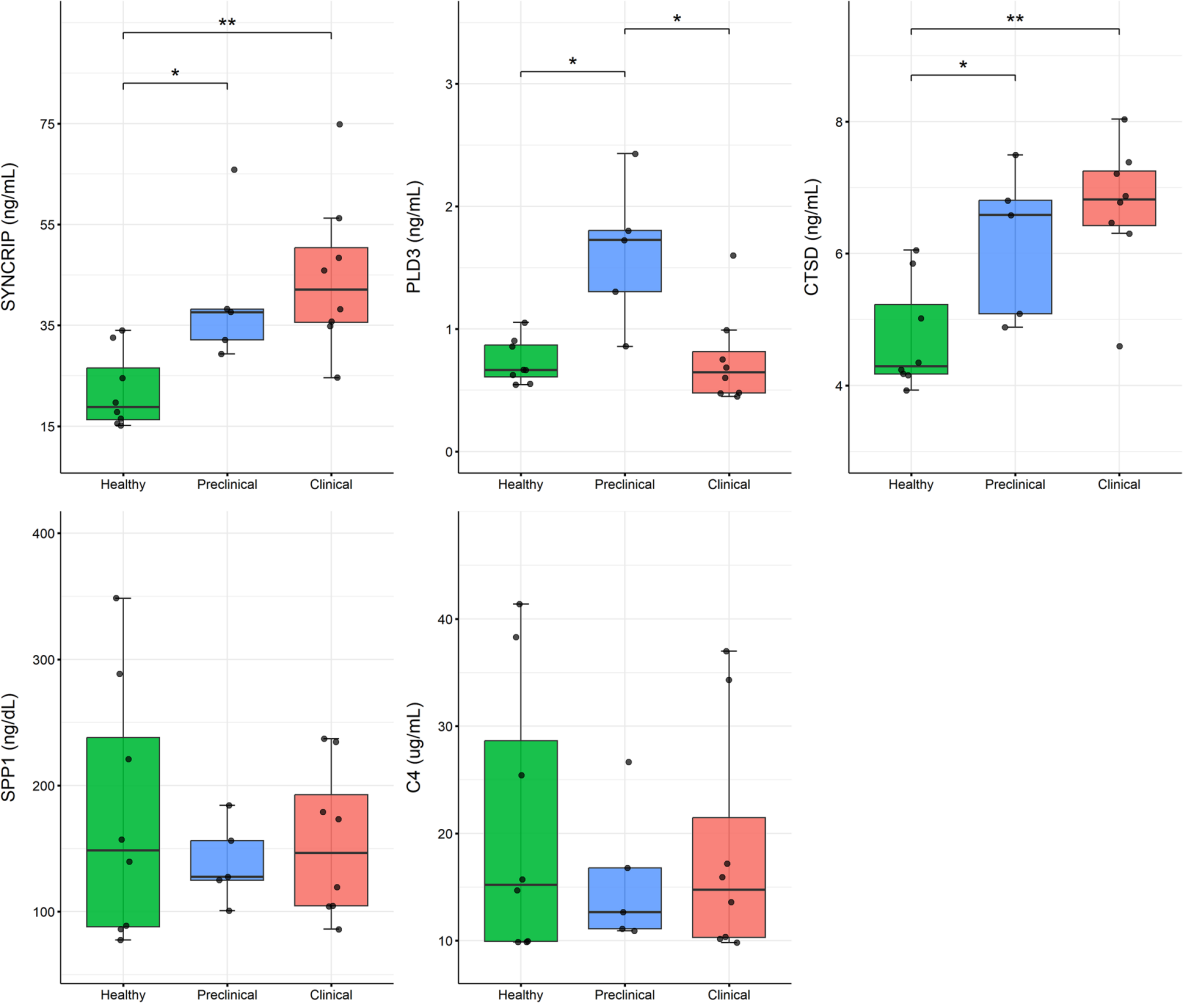
**Serum concentrations of SYNCRIP, PLD3, CTSD, SPP1 and C4 measured by ELISA**. Box plots represent concentrations in healthy controls (green, *n* = 8) and preclinical (blue, *n* = 5) and clinical (red, *n* = 8) scrapie-affected sheep. Boxes span from the first to the third quartile and the median is indicated by the horizontal line inside each box. Whiskers extend to the most extreme data point within 1.5 times the interquartile range; data points beyond this range are considered potential outliers. Statistical significance between groups was assessed using one-way ANOVA test followed by Tukey HSD for normally distributed data, or Kruskal–Wallis followed by Dunn’s test for non-normally distributed data. *p-value < 0.05, **p-value < 0.01.

First, we sought to explore the potential of the five previously CSF-validated proteins (SYNCRIP, PLD3, CTSD, SPP1 and C4) as biomarkers for prion diseases in a more accessible body fluid. To this end, we quantified by ELISA their concentrations in serum from preclinical and clinical scrapie-affected animals, as well as healthy sheep used as controls (Figure 1). This analysis aimed to evaluate whether these proteins could serve as peripheral indicators of disease progression and, more importantly, offer diagnostic value in the early stages of disease prior to the onset of clinical signs.

SYNCRIP concentrations in serum showed a progressive increase throughout the disease progression, with both preclinical (40.6 ± 14.6 ng/mL; *p*-value = 0.049) and clinical (44.9 ± 15.5 ng/mL; *p*-value = 0.006) animals having significantly higher SYNCRIP levels compared to healthy animals (22 ± 7.6 ng/mL). Similarly, CTSD displayed a significantly increased concentration in both preclinical (6.58 ± 1.72 ng/mL; *p*-value = 0.044) and clinical (6.82 ± 0.83 ng/mL; *p*-value = 0.006) scrapie-affected sheep, relative to healthy ones (4.29 ± 1.06 ng/mL). In contrast, PLD3 serum levels showed a distinct pattern, with only preclinical animals (1.73 ± 0.5 ng/mL) showing a significant increase in concentration compared with healthy (0.67 ± 0.26 ng/mL; *p*-value = 0.025) and clinical (0.65 ± 0.34 ng/mL; *p*-value = 0.019) groups. Conversely, SPP1 and C4 failed to show significant differences in serum concentration between groups.

### Neuropathological features throughout scrapie progression

**Figure 2:**
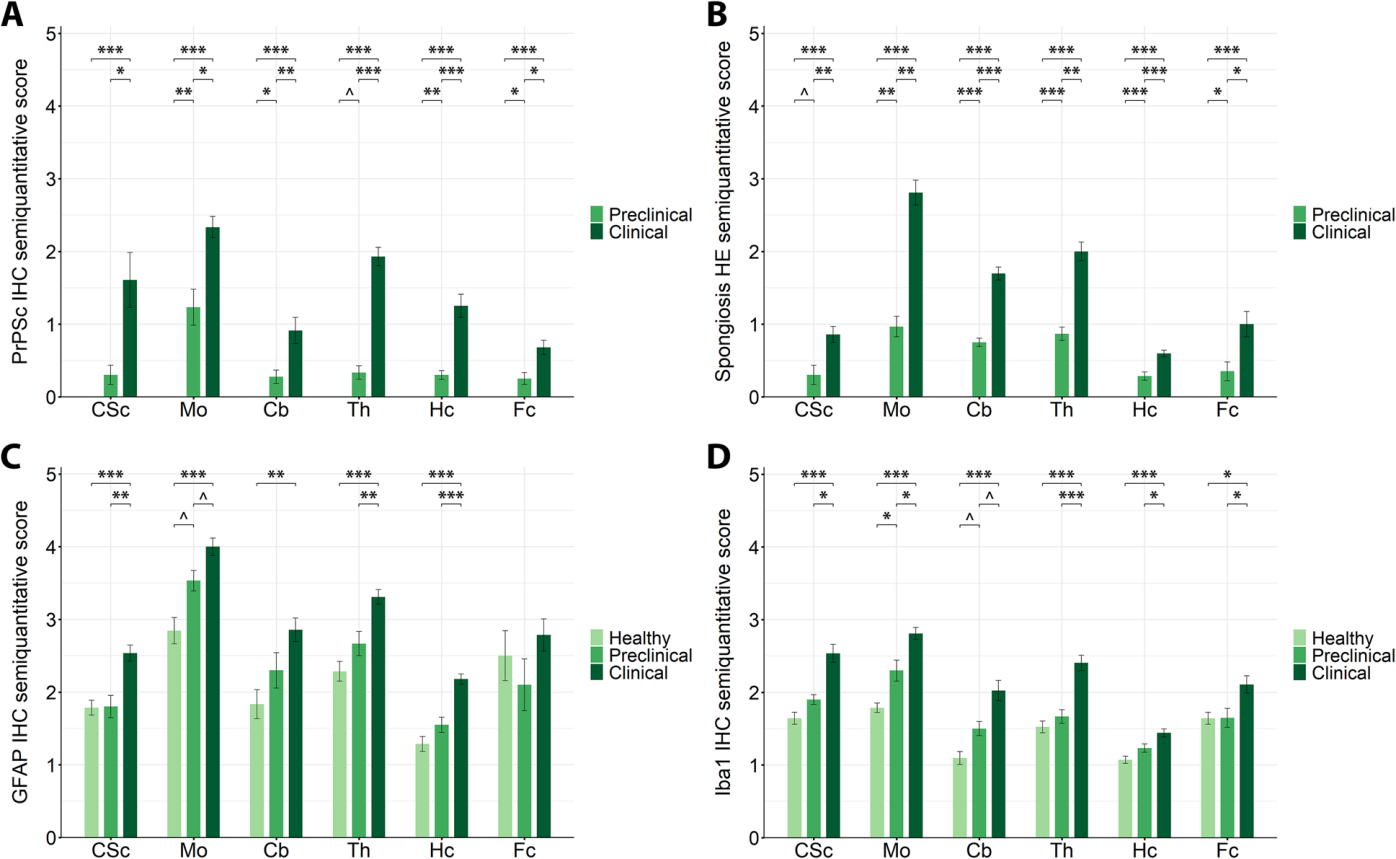
**Neuropathological features in the central nervous system of naturally scrapie-affected sheep**. Bar plots representing semiquantitative score (from 0 to 5) for PrP^Sc^ deposits (PrPSc; **A**), spongiosis (**B**), astrogliosis measured with the glial fibrillary acidic protein (GFAP; **C**) and microgliosis measured using the ionised calcium-binding adaptor molecule 1 (Iba1; **D**), across six brain regions: CSc: cervical spinal cord, Mo: medulla oblongata at the level of the obex, Cb: cerebellum, Th: thalamus, Hc: hippocampus, Fc: frontal cortex. Data presented as the mean and standard deviation. Significant differences between groups were measured using Kruskal–Wallis followed by Dunn’s test. *p-value < 0.05, **p-value < 0.01, ***p-value < 0.001, ^*p*-value < 0.1 (tendency).

We assessed the characteristic neuropathological features of prion diseases in naturally scrapie-affected sheep in both the preclinical and clinical stages of disease, and healthy animals. As shown in Figure 2, the presence of PrP^Sc^ deposits, spongiosis, astrogliosis (GFAP) and microgliosis (Iba1) was evaluated across six CNS regions: cervical spinal cord, medulla oblongata at the level of the obex (hereafter referred to as the obex), cerebellum, thalamus, hippocampus and frontal cortex. As expected, PrP^Sc^ immunostaining was absent in healthy animals and markedly increased in all CNS regions of clinical sheep, with significant accumulation already evident in the preclinical stage, particularly in the obex, cerebellum, hippocampus and frontal cortex, and a close-to-significant increase also observed in the thalamus of preclinical scrapie-affected sheep. Spongiosis followed a similar pattern, showing highly significant differences between scrapie-affected and healthy animals across all regions. Besides, gliosis markers also exhibited region- and stage-dependent differences between groups. Astrogliosis was significantly elevated in clinical animals across most regions, while preclinical sheep only showed a close-to-significant tendency towards increased GFAP immunoreactivity in the obex when compared with healthy sheep. Similarly, microgliosis was significantly increased in the obex of preclinical scrapie-affected sheep, with a close-to-significant increase also observed in the cerebellum of preclinical animals, while it was markedly elevated in the clinical stage in all regions.

### Regional protein distribution, semi-quantification and gene expression of candidate biomarkers

#### Early loss of SYNCRIP immunoreactivity with late gene upregulation in scrapie sheep

**Figure 3:**
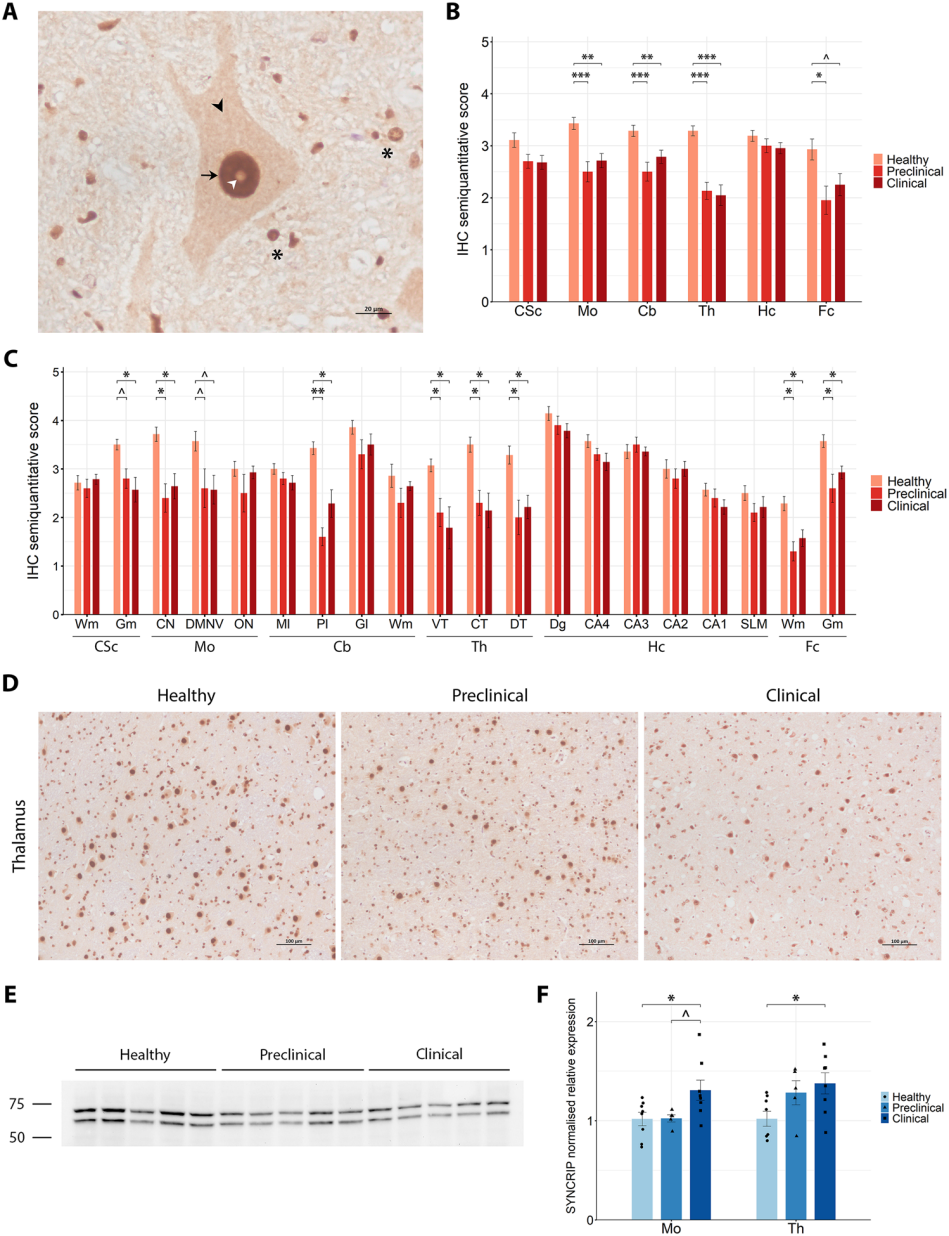
**SYNCRIP expression analysis in the central nervous system of healthy, preclinical and clinical scrapie-affected sheep**. **A** High-magnification image of SYNCRIP immunoreactivity showing uniform intranuclear (arrow), nucleolus-excluding (white arrowhead), and low-intensity cytoplasmic (black arrowhead) staining in neurons, and marked immunostaining in glial cells (asterisks). **B**, **C** Grouped bar plots representing semiquantitative scores (from 0 to 5) of immunohistochemical (IHC) analysis across six brain regions (**B**; CSc: cervical spinal cord, Mo: medulla oblongata at the level of the obex, Cb: cerebellum, Th: thalamus, Hc: hippocampus, Fc: frontal cortex) and their specific subregions (**C**; Wm: white matter, Gm: grey matter, CN: cuneate nucleus, DMNV: dorsal motor nucleus of vagus, ON: inferior olive nucleus, Ml: molecular layer, Pl: Purkinje cell layer, Gl: granular layer, VT: ventral thalamus, CT: central thalamus, DT: dorsal thalamus, Dg: dentate gyrus, CA4-1: cornu ammonis 4–1, SLM: stratum lacunosum-moleculare). Data presented as the mean and standard deviation. Significant differences between groups were measured using Kruskal–Wallis followed by Dunn’s test. *p-value < 0.05, **p-value < 0.01, ***p-value < 0.001, ^*p*-value < 0.1 (tendency). **D** Representative IHC images from healthy, preclinical and clinical sheep in the thalamus. **E** Western blot analysis in thalamic tissue, with molecular weights (in kDa) represented on the left. **F** Grouped bar plot with overlaid scatter plot showing individual 2^−∆∆Ct^ values of gene expression, relative to the healthy group, measured by RT-qPCR in the medulla oblongata at the level of the obex (Mo) and thalamus (Th). Bars and error lines indicate the mean of individual values and standard deviation or the median and interquartile range, for normally and non-normally distributed data, respectively. *SYNCRIP* gene expression was normalised to the mean of *SDHA* and *GAPDH* in the obex and to *SDHA* and *G6PD* in the thalamus. Significant differences between groups were measured using one-way ANOVA test followed by Tukey HSD (for normally distributed data) or Kruskal–Wallis followed by Dunn’s test (for non-normally distributed). *p-value < 0.05, ^*p*-value < 0.1 (tendency).

SYNCRIP immunohistochemical analysis was performed in the same six CNS regions described above, from healthy sheep and sheep naturally affected with scrapie, in both preclinical and clinical stages of the disease. SYNCRIP exhibited an intense, uniform intranuclear immunostaining pattern in neurons, together with a low-intensity neuronal cytoplasmic staining (Figure 3A). Notably, the nucleolus remained consistently unstained across all samples. In addition to its neuronal localisation, SYNCRIP also displayed a marked staining in glial cells. Semi-quantitative analysis of immunostaining intensity revealed a significant SYNCRIP reduction (*p*-value < 0.05) in the obex, cerebellum, thalamus and frontal cortex of scrapie-affected animals compared with healthy controls (Figure 3B). To better locate SYNCRIP alterations between groups, each CNS region was further divided into specific subregions to be analysed (Figure 3C). In both the thalamus and frontal cortex, all examined areas demonstrated significantly reduced SYNCRIP immunoreactivity in scrapie-affected sheep relative to healthy controls. Within the cerebellum, only Purkinje cells exhibited a significant decrease of SYNCRIP staining in scrapie-affected animals compared to healthy ones. Furthermore, several Purkinje cells lacked detectable SYNCRIP staining in scrapie-affected sheep (Additional file 4A). In the cervical spinal cord, immunostaining was less intense in the grey matter of clinical scrapie animals compared with healthy ones, with preclinical animals presenting a close-to-significant tendency towards decreased staining. Representative microscopic images illustrate the progressive decrease in SYNCRIP immunoreactivity from healthy to preclinical and clinical stages (Figure 3D).

WB analysis was performed to confirm the specificity of the SYNCRIP antibody used for the IHC analysis, in the thalamus of five animals per group (Figure 3E). Two distinct bands were observed at approximately 65 kDa and 70 kDa. To complement the findings at protein level, the expression of *SYNCRIP* was assessed by RT-qPCR in the thalamus and obex of the three groups of sheep (Figure 3F). A significant upregulation of SYNCRIP messenger RNA (mRNA) was detected in both the obex (*p*-value = 0.04) and thalamus (*p*-value = 0.036) of clinical scrapie-affected animals compared with healthy controls. Notably, although not significant, a trend towards upregulation can be seen in the preclinical stage in the thalamus.

#### Region-specific PLD3 dysregulation with dynamic protein and gene changes across scrapie progression

**Figure 4:**
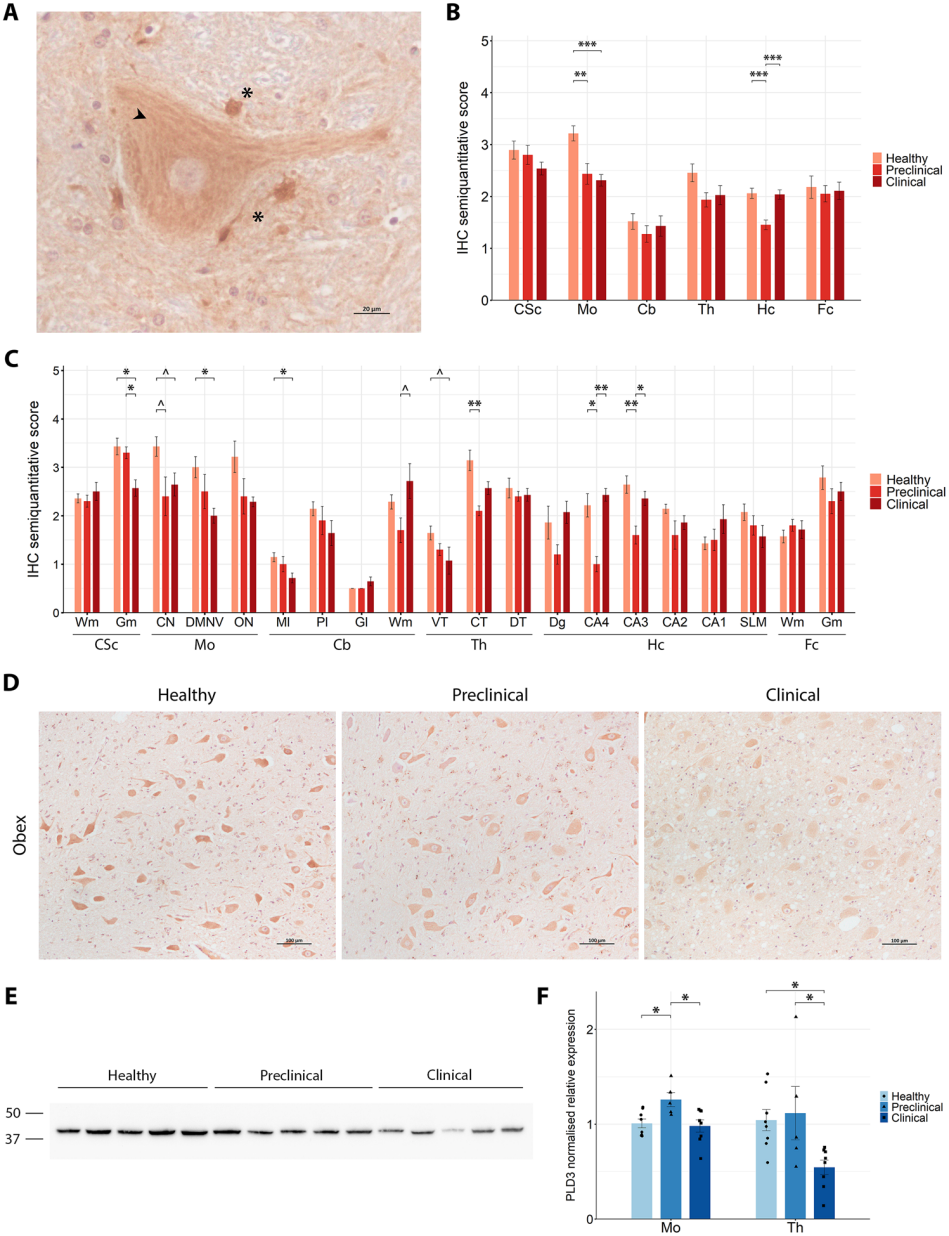
**PLD3 expression analysis in the central nervous system of healthy, preclinical and clinical scrapie-affected sheep**. **A** High-magnification image of PLD3 immunoreactivity showing a striated cytoplasmic staining pattern (arrowhead) in neurons and granular deposits in the neuropil (asterisks). **B**, **C** Grouped bar plots representing semiquantitative scores (from 0 to 5) of immunohistochemical (IHC) analysis across six brain regions (**B**; CSc: cervical spinal cord, Mo: medulla oblongata at the level of the obex, Cb: cerebellum, Th: thalamus, Hc: hippocampus, Fc: frontal cortex) and their specific subregions (**C**; Wm: white matter, Gm: grey matter, CN: cuneate nucleus, DMNV: dorsal motor nucleus of vagus, ON: inferior olive nucleus, Ml: molecular layer, Pl: Purkinje cell layer, Gl: granular layer, VT: ventral thalamus, CT: central thalamus, DT: dorsal thalamus, Dg: dentate gyrus, CA4-1: cornu ammonis 4–1, SLM: stratum lacunosum-moleculare). Data presented as the mean and standard deviation. Significant differences between groups were measured using Kruskal–Wallis followed by Dunn’s test. *p-value < 0.05, **p-value < 0.01, ***p-value < 0.001, ^*p*-value < 0.1 (tendency). **D** Representative IHC images from healthy, preclinical and clinical sheep in the dorsal motor nucleus of vagus of the obex. **E** Western blot analysis in thalamic tissue, with molecular weights (in kDa) represented on the left. **F** Grouped bar plot with overlaid scatter plot showing individual 2^−∆∆Ct^ values of gene expression, relative to the healthy group, measured by RT-qPCR in the medulla oblongata at the level of the obex (Mo) and thalamus (Th). Bars and error lines indicate the mean of individual values and standard deviation or the median and interquartile range, for normally and non-normally distributed data, respectively. *PLD3* gene expression was normalised to the mean of *SDHA* and *GAPDH* in the obex and to *SDHA* and *G6PD* in the thalamus. Significant differences between groups were measured using one-way ANOVA test followed by Tukey HSD (for normally distributed data) or Kruskal–Wallis followed by Dunn’s test (for non-normally distributed). *p-value < 0.05.

PLD3 immunoreactivity was examined across the same six CNS regions, revealing a distinct cytoplasmic distribution in neurons, characterised by a striated pattern, and prominent granular deposits in the neuropil (Figure 4A). Statistical analysis of staining intensity showed a significant reduction in PLD3 deposition in the obex of preclinical and clinical animals compared with healthy controls (Figures 4B, D). In the hippocampus, PLD3 levels showed a marked decrease during the preclinical stage of the disease, with levels returning to those observed in healthy animals during the clinical stage. Detailed analysis of individual areas highlighted significant differences between groups in the dorsal motor nucleus of the vagus in the obex and in the CA3 and CA4 in the hippocampus (Figure 4C). Additionally, reduced PLD3 immunostaining was detected in the central region of the thalamus in preclinical animals compared with healthy controls. In the cervical spinal cord, PLD3 staining intensity in the grey matter was significantly decreased in clinical animals, as previously observed for SYNCRIP.

WB analysis confirmed PLD3 expression at the protein level, revealing a single band at approximately 42 kDa in all groups (Figure 4E). RT-qPCR analysis was then performed to determine the mRNA levels of *PLD3* throughout the progression of the disease, in the same two areas already described: obex and thalamus (Figure 4F). In the obex, preclinical animals exhibited a significant upregulation (*p*-value = 0.036) compared with healthy controls, while clinical animals showed a return to levels comparable to those of the healthy group. Conversely, in the thalamus, *PLD3* expression levels were maintained in preclinical animals, but a significant decrease (*p*-value = 0.044) was observed in the clinical stage of the disease.

#### Reduction of CTSD deposition and early gene upregulation in the regions most affected by scrapie

**Figure 5:**
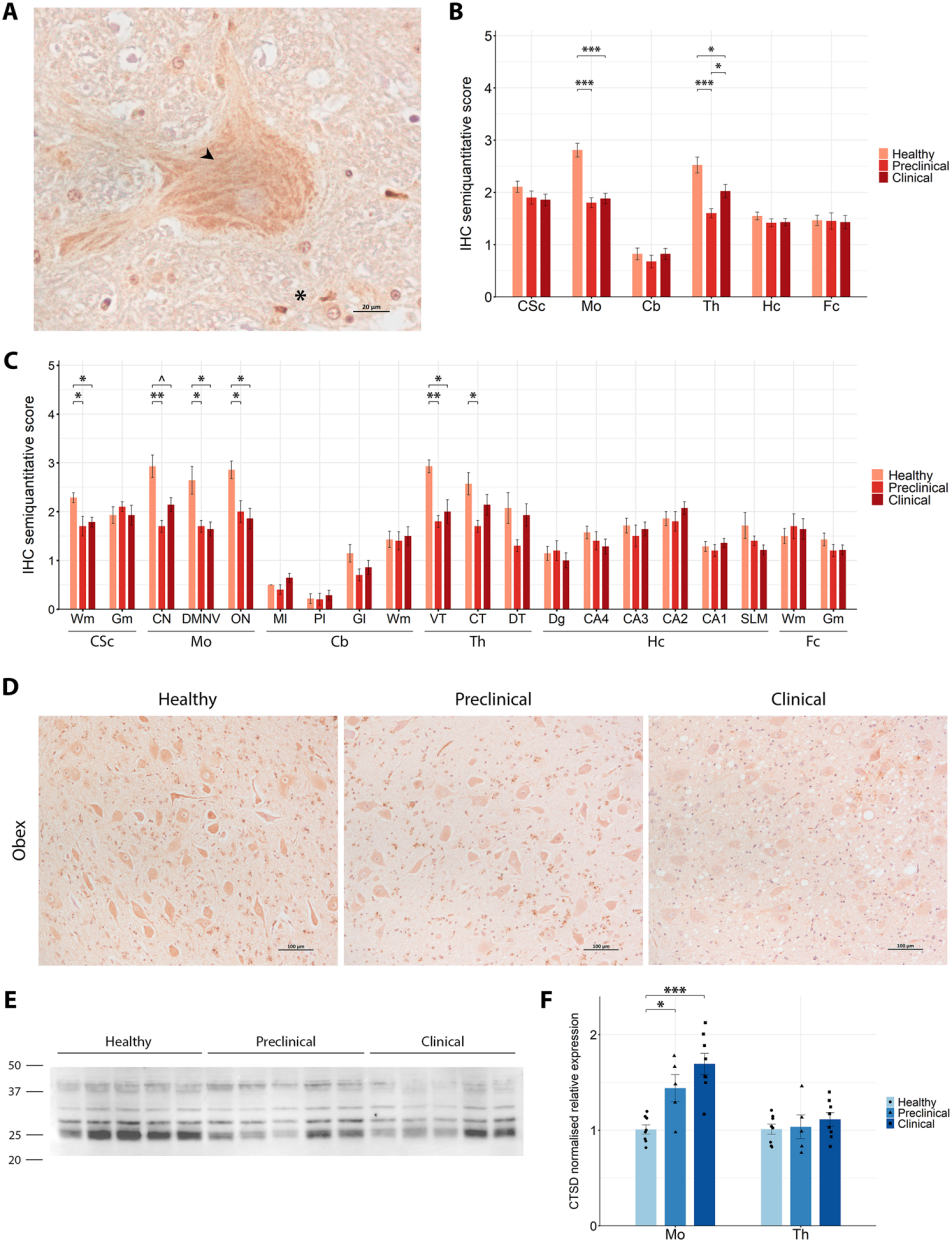
**CTSD expression analysis in the central nervous system of healthy, preclinical and clinical scrapie-affected sheep**. **A** High-magnification image of CTSD immunoreactivity showing a striated cytoplasmic staining pattern (arrowhead) in neurons and granular deposits in the neuropil (asterisk). **B**, **C** Grouped bar plots representing semi-quantitative scores (from 0 to 5) of immunohistochemical (IHC) analysis across six brain regions (**B**; CSc: cervical spinal cord, Mo: medulla oblongata at the level of the obex, Cb: cerebellum, Th: thalamus, Hc: hippocampus, Fc: frontal cortex) and their specific subregions (**C**; Wm: white matter, Gm: grey matter, CN: cuneate nucleus, DMNV: dorsal motor nucleus of vagus, ON: inferior olive nucleus, Ml: molecular layer, Pl: Purkinje cell layer, Gl: granular layer, VT: ventral thalamus, CT: central thalamus, DT: dorsal thalamus, Dg: dentate gyrus, CA4-1: cornu ammonis 4–1, SLM: stratum lacunosum-moleculare). Data presented as the mean and standard deviation. Significant differences between groups were measured using Kruskal–Wallis followed by Dunn’s test. *p-value < 0.05, **p-value < 0.01, ***p-value < 0.001, ^*p*-value < 0.1 (tendency). **D** Representative IHC images from healthy, preclinical and clinical sheep in the dorsal motor nucleus of vagus of the obex. **E** Western blot analysis in thalamic tissue, with molecular weights (in kDa) represented on the left. **F** Grouped bar plot with overlaid scatter plot showing individual 2^−∆∆Ct^ values of gene expression, relative to the healthy group, measured by RT-qPCR in the medulla oblongata at the level of the obex (Mo) and thalamus (Th). Bars and error lines indicate the mean of individual values and standard deviation or the median and interquartile range, for normally and non-normally distributed data, respectively. *CTSD* gene expression was normalised to the mean of *SDHA* and *GAPDH* in the obex and to *SDHA* and *G6PD* in the thalamus. Significant differences between groups were measured using one-way ANOVA test followed by Tukey HSD (for normally distributed data) or Kruskal–Wallis followed by Dunn’s test (for non-normally distributed). *p-value < 0.05, ***p-value < 0.001.

The immunohistochemical analysis of CTSD revealed a pattern closely resembling that of PLD3. A striated cytoplasmic staining was found in neurons, alongside granular deposits in the neuropil (Figure 5A). Notably, only in a subset of healthy sheep, distinct granular cytoplasmic aggregates were observed adjacent to the neuronal nucleus in one of the poles of the perikaryon in specific CNS regions, including the cervical spinal cord grey matter, the hypoglossal and inferior olive nuclei in the obex and the CA in the hippocampus (Additional file 4B). These findings may reflect localised lysosomal aggregation. Semi-quantitative analysis of CTSD immunostaining demonstrated significant differences in staining intensity between groups in two CNS regions. In the thalamus, healthy animals exhibited the highest levels of immunoreactivity, followed by clinical and preclinical sheep, with significant differences (*p*-value < 0.05) among all three groups (Figure 5B). Within specific thalamic areas, both ventral and central regions showed a significant decrease in CTSD staining in preclinical animals compared with healthy ones, and specifically in the ventral thalamus, staining was also significantly reduced in the clinical stage (Figure 5C). In the obex, CTSD immunostaining was significantly reduced in both preclinical and clinical groups compared with healthy animals across all three analysed nuclei, as microscopically represented by the dorsal motor nucleus of the vagus in Figure 5D. A similar reduction was observed in the white matter of the cervical spinal cord, where healthy sheep displayed significantly higher staining intensities than scrapie-affected animals.

WB analysis revealed multiple bands corresponding to different molecular forms of CTSD (Figure 5E). Bands detected at around 38–40 kDa likely represent the precursor preprocathepsin D and procathepsin D. Additionally, a well-defined band at approximately 28 kDa and a thicker band at 25 kDa were observed, which may correspond to the heavy and light chains of the active enzyme. A fainter band was also present at around 32 kDa, potentially representing an intermediate cleavage product. *CTSD* gene expression levels were significantly upregulated in the obex of preclinical (*p*-value = 0.024) and clinical (*p*-value = 1.38 × 10^−4^) scrapie-affected sheep compared with healthy controls (Figure 5F). In the thalamus, however, only a minor, non-significant trend towards upregulation was observed in scrapie-affected animals.

#### SPP1 reduced immunoreactivity with late perineuronal accumulation and regional gene dysregulation

**Figure 6:**
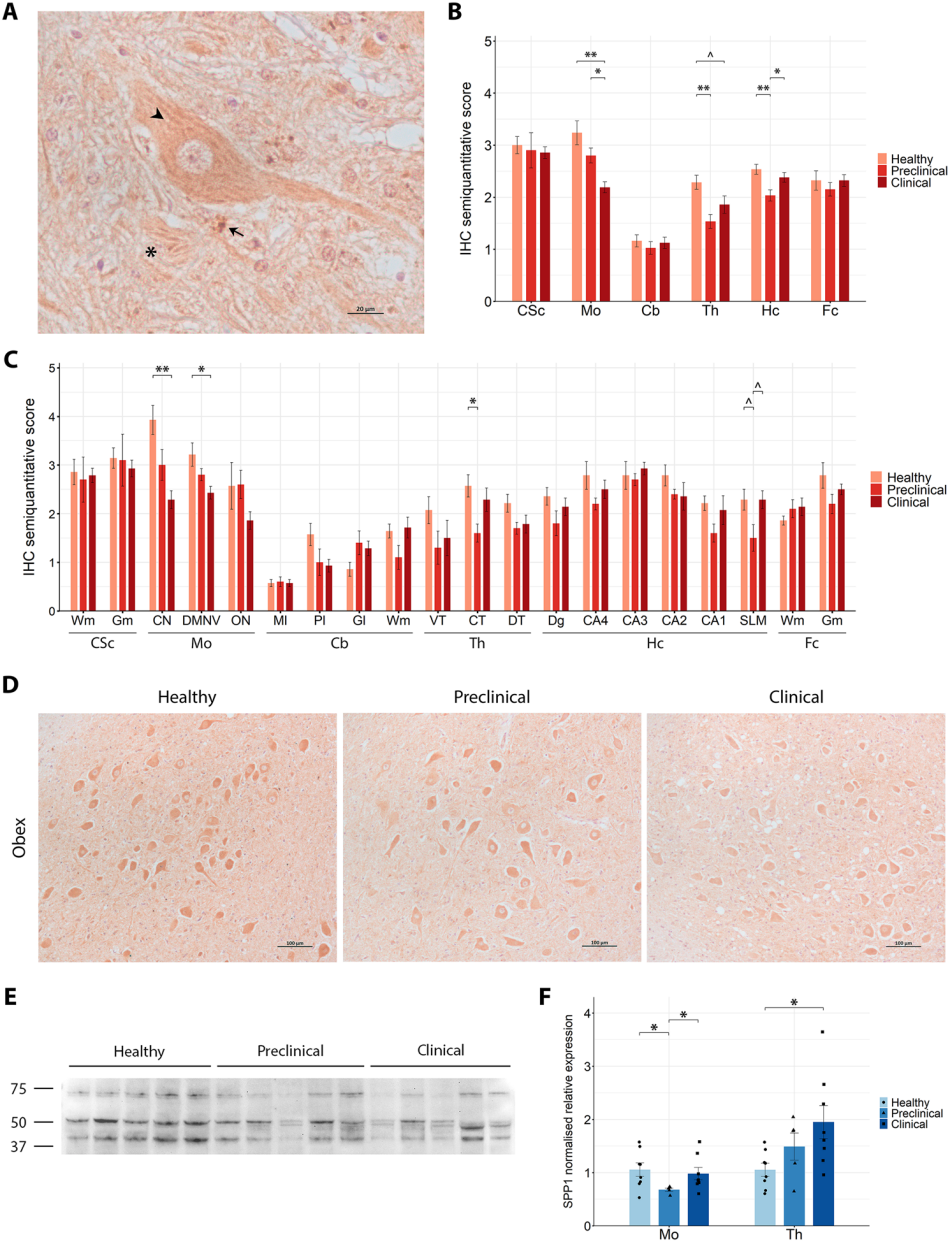
**SPP1 expression analysis in the central nervous system of healthy, preclinical and clinical scrapie-affected sheep**. **A** High-magnification image of SPP1 immunoreactivity showing a striated cytoplasmic staining pattern (arrowhead) in neurons and diffuse filamentous staining (asterisk) with granular deposits (arrow) in the neuropil. **B**, **C** Grouped bar plots representing semiquantitative scores (from 0 to 5) of immunohistochemical (IHC) analysis across six brain regions (**B**; CSc: cervical spinal cord, Mo: medulla oblongata at the level of the obex, Cb: cerebellum, Th: thalamus, Hc: hippocampus, Fc: frontal cortex) and their specific subregions (**C**; Wm: white matter, Gm: grey matter, CN: cuneate nucleus, DMNV: dorsal motor nucleus of vagus, ON: inferior olive nucleus, Ml: molecular layer, Pl: Purkinje cell layer, Gl: granular layer, VT: ventral thalamus, CT: central thalamus, DT: dorsal thalamus, Dg: dentate gyrus, CA4-1: cornu ammonis 4–1, SLM: stratum lacunosum-moleculare). Data presented as the mean and standard deviation. Significant differences between groups were measured using Kruskal–Wallis followed by Dunn’s test. *p-value < 0.05, **p-value < 0.01, ^*p*-value < 0.1 (tendency). **D** Representative IHC images from healthy, preclinical and clinical sheep in the dorsal motor nucleus of vagus of the obex. **E** Western blot analysis in thalamic tissue, with molecular weights (in kDa) represented on the left. **F** Grouped bar plot with overlaid scatter plot showing individual 2^−∆∆Ct^ values of gene expression, relative to the healthy group, measured by RT-qPCR in the medulla oblongata at the level of the obex (Mo) and thalamus (Th). Bars and error lines indicate the mean of individual values and standard deviation or the median and interquartile range, for normally and non-normally distributed data, respectively. *SPP1* gene expression was normalised to the mean of *SDHA* and *GAPDH* in the obex and to *SDHA* and *G6PD* in the thalamus. Significant differences between groups were measured using one-way ANOVA test followed by Tukey HSD (for normally distributed data) or Kruskal–Wallis followed by Dunn’s test (for non-normally distributed). *p-value < 0.05.

SPP1 immunohistochemical analysis showed a predominantly striated cytoplasmic staining pattern in neurons, similar to that observed for PLD3 and CTSD (Figure 6A). In the neuropil, a diffuse and filamentous staining, likely corresponding to neuronal processes, was the predominant pattern, together with occasional granular deposits. Interestingly, a perineuronal immunostaining, consistent with labelling of the neuronal membrane, was observed in the mesencephalon of a subset of clinical sheep (Additional file 4C). Semi-quantitative score analysis identified significant differences in SPP1 immunostaining between groups in several regions (Figure 6B). In the obex, a decrease of SPP1 was observed in scrapie-affected sheep, starting with a trend in preclinical animals which became significant in the clinical stage compared with healthy ones. In the thalamus, a significant reduction in staining intensity was observed in preclinical animals compared with healthy controls, with clinical animals displaying a slight, close-to-significant decrease. Besides, SPP1 staining intensity was significantly reduced in the hippocampus at the preclinical stage of the disease, compared with both healthy and clinical animals, with the latter returning to levels comparable to those of healthy controls. When specific areas within these regions were analysed (Figure 6C), both the cuneate nucleus and the dorsal motor nucleus of the vagus in the obex demonstrated significant reductions in staining in clinical animals, represented in Figure 6D. Additionally, the central area of the thalamus and the SLM of the hippocampus were the main contributors to the overall significance of their respective regions.

WB identified three bands at approximately 70 kDa, 50 kDa and 40 kDa (Figure 6E). Notably, the 50-kDa band appeared as a doublet in some preclinical and clinical scrapie-affected animals, whereas it was a single band in healthy controls. This pattern variation suggests potential post-translational modifications or differential processing of SPP1 in different disease states. RT-qPCR gene expression analysis revealed a significant downregulation of *SPP1* in the obex of preclinical animals compared with both healthy and clinical groups, with clinical animals displaying mRNA levels nearly equivalent to those of healthy sheep (Figure 6F). Conversely, in the thalamus, while preclinical scrapie-affected animals showed a trend towards upregulation of *SPP1*, a significant upregulation (*p*-value = 0.033) was evident in the clinical stage of the disease, compared to healthy controls.

#### Early cerebellar loss of C4 immunoreactivity and progressive upregulation in the regions most affected by scrapie

**Figure 7:**
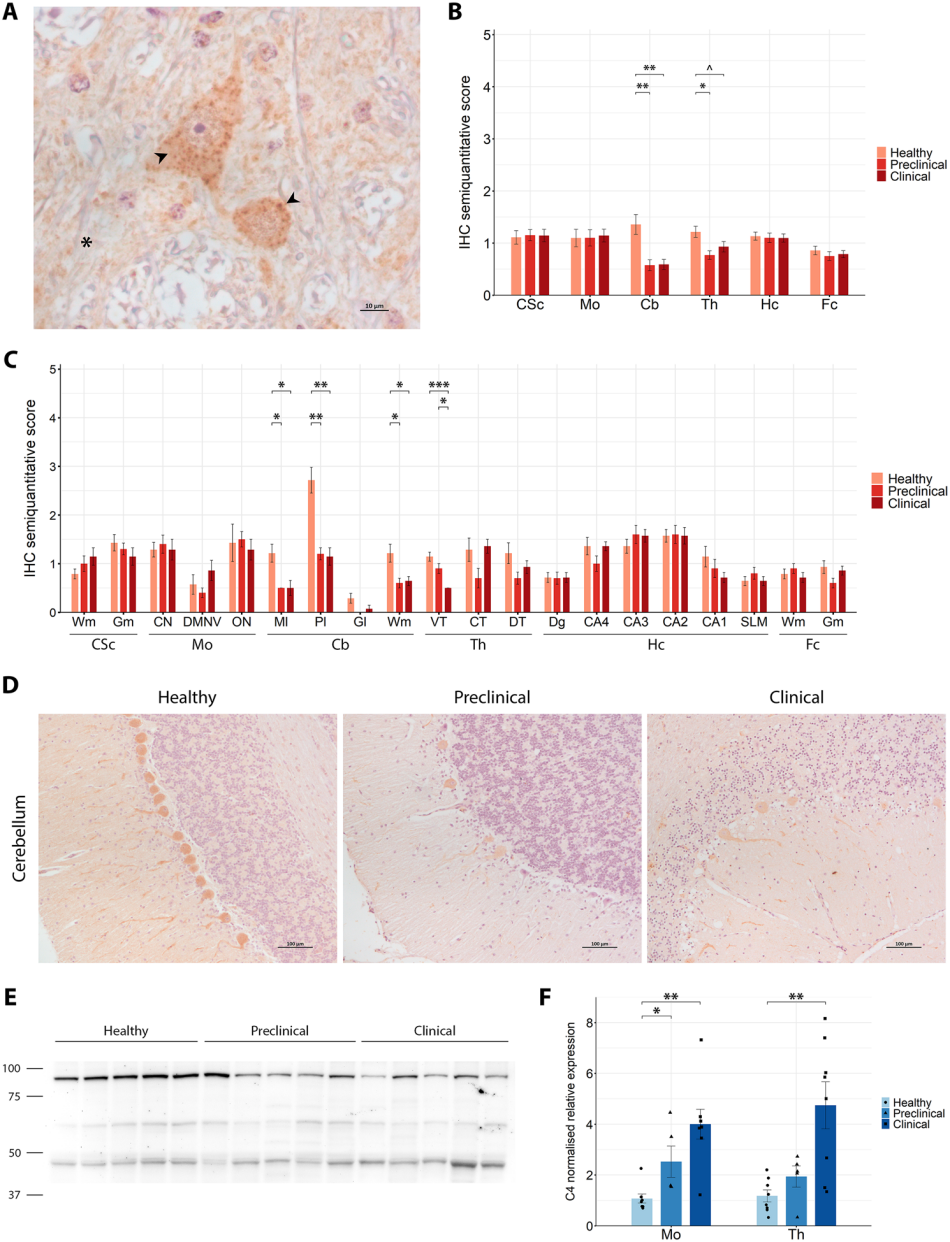
**C4 expression analysis in the central nervous system of healthy, preclinical and clinical scrapie-affected sheep**. **A** High-magnification image of C4 immunoreactivity showing a granular cytoplasmic staining pattern (arrowheads) in neurons and low-intensity diffuse staining in the neuropil (asterisk). **B**, **C** Grouped bar plots representing semiquantitative scores (from 0 to 5) of immunohistochemical (IHC) analysis across six brain regions (**B**; CSc: cervical spinal cord, Mo: medulla oblongata at the level of the obex, Cb: cerebellum, Th: thalamus, Hc: hippocampus, Fc: frontal cortex) and their specific subregions (**C**; Wm: white matter, Gm: grey matter, CN: cuneate nucleus, DMNV: dorsal motor nucleus of vagus, ON: inferior olive nucleus, Ml: molecular layer, Pl: Purkinje cell layer, Gl: granular layer, VT: ventral thalamus, CT: central thalamus, DT: dorsal thalamus, Dg: dentate gyrus, CA4-1: cornu ammonis 4–1, SLM: stratum lacunosum-moleculare). Data presented as the mean and standard deviation. Significant differences between groups were measured using Kruskal–Wallis followed by Dunn’s test. *p-value < 0.05, **p-value < 0.01, ***p-value < 0.001, ^*p*-value < 0.1 (tendency). **D** Representative IHC images from healthy, preclinical and clinical sheep in the cerebellum. **E** Western blot analysis in thalamic tissue, with molecular weights (in kDa) represented on the left. **F** Grouped bar plot with overlaid scatter plot showing individual 2^−∆∆Ct^ values of gene expression, relative to the healthy group, measured by RT-qPCR in the medulla oblongata at the level of the obex (Mo) and thalamus (Th). Bars and error lines indicate the mean of individual values and standard deviation or the median and interquartile range, for normally and non-normally distributed data, respectively. *C4* gene expression was normalised to the mean of *SDHA* and *GAPDH* in the obex and to *SDHA* and *G6PD* in the thalamus. Significant differences between groups were measured using one-way ANOVA test followed by Tukey HSD (for normally distributed data) or Kruskal–Wallis followed by Dunn’s test (for non-normally distributed). *p-value < 0.05, **p-value < 0.01.

C4 immunoreactivity was observed predominantly in the cytoplasm of neurons, with a defined granular pattern, alongside a less-intense, diffuse staining in the neuropil, in all groups of sheep (Figure 7A). Semi-quantitative evaluation of C4 immunostaining revealed generally low intensities, with scores between 0 and 2 on the 0–5 scale. Despite the overall low immunoreactivity levels, significant differences were observed in the cerebellum and thalamus (Figure 7B). In the cerebellum, scrapie-affected animals at both stages of the disease exhibited a significant decrease in C4 staining compared with healthy controls. Among cerebellar subregions, this reduction was observed across the molecular, Purkinje cell and white matter layers, whereas the granular layer showed imperceptible staining (Figure 7C). Representative cerebellar microscopic images are provided in Figure 7D. In the thalamus, preclinical animals showed a significant reduction in immunostaining compared with healthy controls, while a close-to-significant tendency towards decreased staining was found in the clinical stage. Within thalamic subregions, only the ventral area displayed statistically significant differences.

WB analysis detected three bands at approximately 90 kDa, 60 kDa and 46 kDa in all samples (Figure 7E). These bands likely correspond to the three polypeptide chains of C4-alpha, beta and gamma. RT-qPCR analysis showed a significant fourfold upregulation of *C4* mRNA levels in clinical animals compared with healthy controls in the obex (*p*-value = 0.001), and an almost fivefold upregulation in the thalamus (*p*-value = 0.009) (Figure 7F). Additionally, significant upregulation of *C4* was specifically observed in the obex at the preclinical stage of disease when compared with healthy controls.

### Protein expression significantly correlates with prion-associated neuropathology

**Figure 8:**
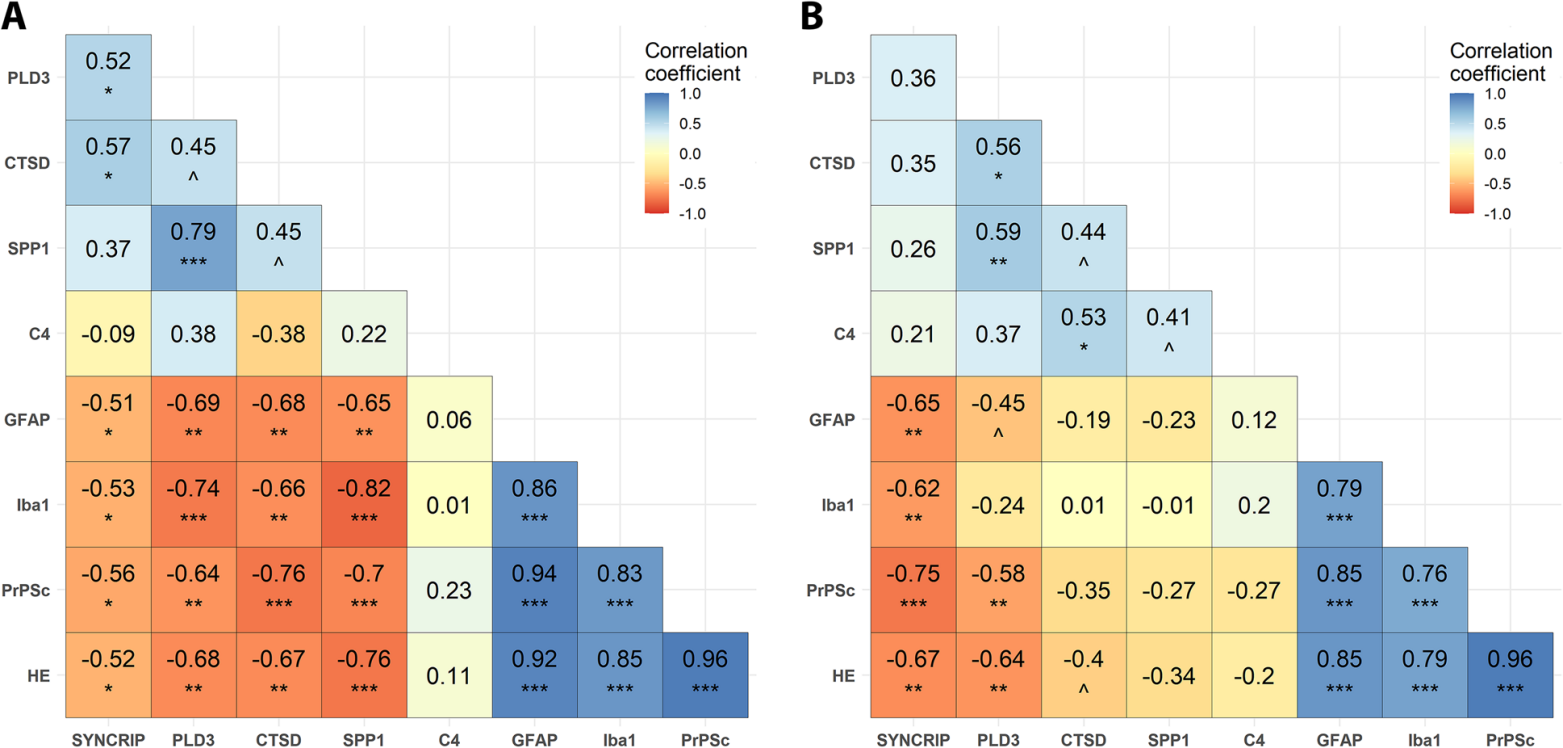
**Correlation matrix between SYNCRIP, PLD3, CTSD, SPP1 and C4 and prion neuropathology, in obex** (**A**) **and thalamus** (**B**). Spearman correlation coefficients and significance between the semiquantitative scores of immunohistochemical analysis of the five proteins in healthy (*n* = 7) and preclinical (*n* = 5) and clinical (*n* = 7) naturally scrapie-affected sheep and their neuropathological scores: PrP^Sc^ deposits (PrPSc), spongiosis (HE), astrogliosis measured with the glial fibrillary acidic protein (GFAP), and microgliosis measured using the ionised calcium-binding adaptor molecule 1 (Iba1). *p-value < 0.05, **p-value < 0.01, ***p-value < 0.001, ^*p*-value < 0.1 (tendency).

To explore potential relationships between protein immunoreactivity and neuropathological alterations, we performed correlation analyses between the semiquantitative IHC scores of the five proteins and the key neuropathological features of prion diseases: PrP^Sc^ deposition, spongiosis, astrogliosis and microgliosis. These correlations were assessed in the obex (Figure 8A) and thalamus (Figure 8B), the two CNS regions most affected by scrapie and where the most significant differences between groups were observed in the IHC of the analysed proteins. As expected, all neuropathological markers showed strong and highly significant correlations among themselves (*p*-value < 0.001) in both regions.

In the obex, SYNCRIP, PLD3, CTSD and SPP1 exhibited significant negative correlations with all neuropathological features, indicating that lower protein levels were associated with more severe prion-related neuropathology, in line with the IHC results. While SYNCRIP showed slightly weaker correlations, PLD3, CTSD and SPP1 displayed stronger associations with neuropathology in this CNS region. Besides, in the thalamus, SYNCRIP demonstrated the strongest and most significant negative correlations with all neuropathological markers. PLD3 also correlated significantly with PrP^Sc^ and spongiosis, but showed no significant association with microgliosis, and only a close-to-significant tendency with astrogliosis. Regarding inter-protein correlations, PLD3 and SPP1 exhibited significant positive correlations in both regions. Additionally, a close-to-significant positive correlation was found between PLD3 and CTSD in the obex, which turned significant in the thalamus.

On the other hand, C4 failed to correlate with neuropathology in the two CNS regions analysed. However, when we performed this correlation analysis in the cerebellum, the region in which C4 showed higher expression and most significant differences between groups in the IHC analysis, its protein levels significantly correlated with the neuropathological features (Additional file 5).

## Discussion

Using naturally scrapie-affected sheep as a preclinical model for prion diseases, this study provides new evidence on the diagnostic potential of five previously CSF-validated proteins, by exploring their concentration in serum and their expression across different disease stages and their relationship with prion neuropathology. The most remarkable finding of this study is the significant increase in serum levels of SYNCRIP, PLD3 and CTSD at the preclinical stage of scrapie, reinforcing the potential of these proteins as early, easily accessible diagnostic biomarkers for prion diseases and highlighting serum as a promising peripheral body fluid for early disease detection. Additionally, IHC and RT-qPCR revealed significant alterations in both protein and gene expression, in most cases preceding the onset of clinical signs, particularly in the obex and thalamus, two key regions affected in scrapie. These changes correlated strongly with neuropathological features, reinforcing their potential role in disease progression.

Despite its well-documented role in RNA metabolism, research on SYNCRIP (or hnRNP Q) in neurodegenerative diseases remains scarce, particularly in prion diseases. The detection of significantly elevated SYNCRIP levels in the serum of both preclinical and clinical scrapie-affected animals provides the first evidence of its potential as an easily accessible biomarker in prion diseases, mirroring its behaviour in CSF reported in our previous study [9]. Beyond serum, our findings also offer the first evidence of SYNCRIP dysregulation at both protein and gene expression levels across different CNS regions affected by natural scrapie, with changes detected as early as the preclinical stage. Notably, SYNCRIP immunostaining was reduced in scrapie-affected animals in the obex, thalamus and frontal cortex, with an evident loss in Purkinje cells, suggesting a potential role in cerebellar dysfunction. This aligns with previous research from Bakkar and colleagues, who found SYNCRIP immunoreactivity particularly altered in the cerebellum of patients with ALS, although in that case, protein levels were increased compared with healthy controls [23]. However, they found an upregulation of *SYNCRIP* mRNA in motor neurons, which is in accordance with our significant upregulation detected in the two most affected CNS regions in the clinical stage. Interestingly, our WB revealed two bands, which may correspond to different SYNCRIP isoforms, as previously described [24], or closely related heterogeneous nuclear ribonucleoproteins (hnRNP) family members, such as hnRNP R, given their high sequence homology. The presence of these isoforms could explain the nuclear and cytoplasmic distribution observed in our immunostaining, which is consistent with previous findings [24, 25]. This reinforces the dynamic nature of hnRNP proteins, known to shuttle between cellular compartments to regulate RNA metabolism [26]. While the precise function of SYNCRIP in prion diseases remains unclear, the early and widespread alterations observed here suggest a role in disease progression or neuroprotection. Given its recently identified involvement in neurogenesis [27], the upregulation of *SYNCRIP* mRNA in scrapie-affected regions may partly represent a compensatory response to neuronal loss.

Additionally, PLD3 and CTSD are lysosomal proteins involved in protein degradation and apoptosis, essential processes in neurodegeneration [28, 29]. Both proteins showed significant alterations in serum at the preclinical stage of scrapie, with PLD3 levels returning to baseline in clinical animals, mirroring the biphasic profile previously observed for CTSD in CSF [9]. These findings suggest a complex and potentially complementary regulation of these lysosomal proteins in peripheral body fluids during prion disease progression. Besides, the significant correlation observed between PLD3 and CTSD in the thalamus, together with the close-to-significant correlation in the obex and their similar immunostaining patterns, suggests a shared response to prion infection and neurodegeneration. Additionally, both proteins colocalise and interact in neuronal lysosomes in AD brains [30, 31] and are implicated in beta-amyloid degradation [32, 33]. Interestingly, the preclinical pattern observed for CTSD in the CSF [9], where levels increased in preclinical animals before returning to basal levels in the clinical stage, is now mirrored by PLD3 in this study. *PLD3* upregulation was detected at the gene level in the obex during the preclinical stage, as well as at the protein level in serum, where its concentration was elevated in the preclinical stage but returned to normal levels in clinical animals. We can hypothesise that *PLD3* may initially be upregulated in response to prion-induced stress in the preclinical stage and the compensatory mechanisms may be unsustainable when lysosomal dysfunction worsens, which was not evident in its CSF profile. However, PLD3 immunostaining was reduced in the obex in both preclinical and clinical animals, and its mRNA levels were downregulated in the thalamus in clinical sheep. This aligns with PLD3 downregulation reported at both protein and gene levels in AD brains [34, 35]. Conversely, *CTSD* mRNA levels showed a progressive and significant increase throughout disease progression in the obex, consistent with its upregulation in scrapie-infected mouse brains and sCJD cases [36–38]. This may be a mechanism to counteract low protein levels, seen in scrapie-affected sheep mainly in the obex and thalamus, as its deficiency has been linked to protein aggregate propagation in alpha-synucleinopathies [39]. Serum protein levels were also elevated in both preclinical and clinical animals, differing from previous reports on reduced CTSD in plasma of patients with AD and PD [40, 41]. The contrasting regulation of these proteins raises questions about the underlying mechanisms driving protein redistribution across different compartments during prion pathogenesis. PLD3 and CTSD may have different dynamics in lysosomal release and degradation, depending on the tissue and disease stage, but further research is needed to clarify these processes.

Furthermore, SPP1 and C4 both play key roles in neuroinflammation, being involved in immune signalling and synaptic refinement [42, 43]. In contrast to the significant changes found in CSF for both proteins [9], serum concentrations of SPP1 and C4 did not show significant differences between healthy and scrapie-affected animals. This discrepancy may be attributed to the systemic nature of these proteins, as they both play key roles in peripheral inflammation and immune responses beyond the CNS. Elevated SPP1 in serum has been reported in ALS [44], though other studies have found no significant differences between diseased and healthy individuals [45]. Similarly, studies on serum C4 in neurodegenerative diseases have shown variable results, with increased levels reported in ALS [46] but no differences or even decreased concentrations in AD [47]. These conflicting findings, along with the lack of significant serum changes in our study, suggest that peripheral inflammatory processes may mask CNS-specific alterations that are more clearly detectable in CSF, a challenge already noted in schizophrenia research on C4 [48]. Despite these negative results in serum, gene expression analyses showed an upregulation of *SPP1* expression in the thalamus during the clinical stage, despite its early downregulation in the obex, and a marked increase in *C4* mRNA levels in both the obex and thalamus of clinical sheep, significant even at the preclinical stage in the obex. These results align with previous studies demonstrating increased *SPP1* expression in sCJD brains [38, 49], scrapie-infected mice [50] and PD microglia [51], alongside *C4* upregulation in sCJD [52] and AD brains [53], with complement activation contributing to neuroinflammation and synaptic dysfunction [54, 55]. However, at the protein level, both SPP1 and C4 showed decreased immunoreactivity in the CNS, which contrasts with some previous findings. For instance, SPP1 protein levels were reported to be increased in the AD brain [56]. Nevertheless, SPP1 release is known to serve as a signal for microglial activation, and in this study, its decrease—or most probably its release from neurons—appears to be most prominent in the obex and thalamus, regions with the highest microglial activation in scrapie-affected animals [57]. Besides, Llorens and colleagues reported increased C4 protein levels in sCJD frontal cortex and cerebellum [52], with elevated C4 levels also described in the substantia nigra from PD mice [58]. The reduced C4 protein levels seen here, particularly in Purkinje cells, could suggest complement consumption or altered processing within these neurons.

Interestingly, our findings revealed strong significant correlations between the expression of the five proteins analysed and neuropathological markers (PrP^Sc^ deposition, spongiosis, astrogliosis and microgliosis). Notably, correlations were more evident in the obex, suggesting that these proteins may be particularly relevant in early prion pathology, as the obex is known to be a primary site for PrP^Sc^ accumulation in natural scrapie and one of the most severely affected areas [16, 59]. A potential limitation of this study is the relatively small sample size, partly due to the use of the natural disease model, which also leads to possible differences in the pathological stage of animals within each group. Another limitation is the cross-sectional design of the study that prevents tracking disease progression within individuals. Nevertheless, the consistency of the correlations provides promising insights into protein alterations throughout the stages of the disease. Future longitudinal studies would further validate these results and clarify the sequential progression of the molecular changes observed.

Moreover, the results of this study provide preliminary answers to the hypothesis posed in our previous work [9] regarding the mechanisms underlying protein overexpression in the CSF of naturally scrapie-affected animals. The observed increase in CSF protein levels in diseased animals, also mirrored here in serum, is likely due in part to the release of proteins from degenerating neurons, as supported by the reduced immunostaining observed in the CNS from the preclinical stage of disease onwards. However, our significant RT-qPCR results also suggest a pathological upregulation of gene expression, indicating that the CNS may be actively increasing protein synthesis in response to disease progression, and that the elevated levels in the CSF are not solely due to neuronal loss. Notably, the recurrent observation of increased mRNA expression concomitant with reduced CNS protein immunoreactivity across several candidates underscores the importance of validating transcriptomic alterations at the protein level and highlights the potential involvement of shared disease-associated mechanisms acting downstream of transcription and contributing to altered protein expression and distribution across compartments. Nevertheless, discrepancies with previous studies reporting different patterns of protein expression in the CNS suggest that further research is needed to clarify whether these findings are specific to scrapie or reflect broader prion disease mechanisms. Additionally, the role of neuronal loss in modulating protein levels requires further exploration to determine its impact on the redistribution of these proteins between the CNS and body fluids.

In conclusion, our findings highlight the promising role of SYNCRIP, PLD3 and CSTD as preclinical easily accessible diagnostic biomarkers of prion diseases in highly accessible body fluids such as serum, complementing previous evidence obtained in CSF. Moreover, all five proteins analysed (SYNCRIP, PLD3, CTSD, SPP1 and C4) exhibit significant expression differences in CNS across disease stages, closely associated with prion-induced neuropathology, further supporting their relevance in disease progression. Further research is warranted to validate our results and study the potential prognostic and therapeutic implications of these proteins in prion and other neurodegenerative diseases.

## Supplementary Information

**Additional file 1.** **Immunohistochemical control sections**. Representative images from healthy and scrapie-affected sheep brain sections processed under identical conditions as experimental samples, in which primary antibodies were substituted with species-matched IgG isotype control. No immunoreactivity was detected for any of the antibodies analysed.

**Additional file 2.** **Primers used for real-time quantitative PCR gene expression analysis**.

**Additional file 3.** **RefFinder results for the potential housekeeping genes analysed, in obex (A) and thalamus (B)**. Ranking of the overall stability of the potential housekeeping genes, calculated based on the geometric mean of ranking values, derived from multiple computational algorithms.

**Additional file 4.** **Distinct immunohistochemical staining patterns of SYNCRIP, CTSD and SPP1 in specific central nervous system regions**. **A** High-magnification immunohistochemical (IHC) images of the cerebellar cortex showing SYNCRIP distribution in Purkinje cells (asterisks) from healthy, preclinical and clinical scrapie-affected sheep. Marked immunoreactivity is observed in healthy Purkinje cells, while a complete loss of staining is evident in scrapie-affected Purkinje cells. **B** Representative images from healthy animals showing distinct granular cytoplasmic aggregates (arrowheads) of CTSD in several central nervous system regions (left to right): cervical spinal cord grey matter (Gm), hypoglossal nucleus (HN) of the obex, inferior olive nucleus (ON) of the obex, and *Cornu Ammonis* 2 (CA2) of the hippocampus. **C** IHC detection of SPP1 in the obex of clinically affected animals, highlighting perineuronal immunoreactivity (arrows) in neurons.

**Additional file 5.** **Correlation matrix between SYNCRIP, PLD3, CTSD, SPP1 and C4 and prion neuropathology, in the cerebellum**. Spearman correlation coefficients and significance between the semiquantitative scores of immunohistochemical analysis of the five proteins in healthy (*n* = 7) and preclinical (*n* = 5) and clinical (*n* = 7) naturally scrapie-affected sheep and their neuropathological scores: PrP^Sc^ deposits (PrPSc), spongiosis (HE), astrogliosis measured with the glial fibrillary acidic protein (GFAP), and microgliosis measured using the ionised calcium-binding adaptor molecule 1 (Iba1). *p-value < 0.05, **p-value < 0.01, ***p-value < 0.001, ^*p*-value < 0.1 (tendency).

## Data Availability

No datasets were generated or analysed during the current study.

## Abbreviations

AD: Alzheimer’s disease
ALS: amyotrophic lateral sclerosis
BSE: bovine spongiform encephalopathy
C4: complement component 4
CA: cornu ammonis
cDNA: complementary DNA
CNS: central nervous system
CSF: cerebrospinal fluid
Ct: cycle threshold
CTSD: cathepsin D
DAB: diaminobenzidine
ELISA: enzyme-linked immunosorbent assay
G6PD: glucose-6-phosphate dehydrogenase
GAPDH: glyceraldehyde-3-phosphate dehydrogenase
GFAP: glial fibrillary acidic protein
HE: haematoxylin-eosin
hnRNP: heterogeneous nuclear ribonucleoproteins
Iba1: ionised calcium binding adaptor molecule 1
IHC: immunohistochemistry
mRNA: messenger RNA
PD: Parkinson’s disease
PLD3: phospholipase D family member 3
PMCA: protein misfolding cyclic amplification
PrP^C^: cellular prion protein
PrP^Sc^: pathological prion protein
RT: room temperature
RT-qPCR: real-time quantitative polymerase chain reaction
RT-QuIC: real-time quaking-induced conversion
sCJD: sporadic Creutzfeldt–Jakob disease
SDHA: succinate dehydrogenase complex flavoprotein subunit A
SLM: stratum lacunosum-moleculare
SPP1: osteopontin
SYNCRIP: synaptotagmin binding, cytoplasmic RNA interacting protein
TBST: Tris-buffered saline with 0.1% Tween 20
TSE: transmissible spongiform encephalopathies
WB: western blot
