# Supplementary material for: Preclinical serum alterations and tissue changes in protein and gene expression of early cerebrospinal fluid-validated biomarkers in scrapie
Source: Vet Res. 2026 Jun 2;57:98. doi: 10.1186/s13567-026-01759-1 (PMC13231744; doi:10.1186/s13567-026-01759-1)
Supplement: Supplementary file 2 — Additional file 2. Primers used for real-time quantitative PCR gene expression analysis. [file 13567_2026_1759_MOESM2_ESM.docx]

Additional file 2. **Primers used for real-time quantitative PCR gene expression analysis.**

| **Gene** | **Forward (F) and reverse (R) primer sequences (5’🡪3’)** | **Concentration (nM)** | **Amplicon length (bp)** | **GenBank accession number** |
| --- | --- | --- | --- | --- |
| *GAPDH*^1^ | F: TCCATGACCACTTTGGCATCGT  R: GTCTTCTGGGTGGCAGTGA | 900 | 80 | NM_001190390.1 |
| *G6PD*^1^ | F: TGACCTATGGCAACCGATACAA  R: CCGCAAAAGACATCCAGGAT | 300 | 76 | NM_001093780.1 |
| *SDHA*^1^ | F: CATCCACTACATGACGGAGCA  R: ATCTTGCCATCTTCAGTTCTGCTA | 300 | 90 | XM_027980212.2 |
| *SYNCRIP* | F: TCAGACATTGCTTGATGCTGG  R: TGCTGAAGAACTGCCAATGC | 300 | 156 | XM_004011296.6 |
| *PLD3* | F: TCAATGGAACCCCTGCTCTG  R: ATGAAACTCCGGGCACTGTC | 500 | 118 | XM_042232056.1 |
| *SPP1* | F: CACAAATGATGGCCGAGGTG  R: TAGGTGCGTCATGCATCTCC | 300 | 146 | NM_001009224.1 |
| *CTSD* | F: TGTCTTCGACAACCTGATGC  R: AGGCTGCCTCTGTAGTACTTG | 400 | 138 | XM_027959254.2 |
| *C4* | F: TCCCTGGGAAGCAAGATTAACG  R: CATGACATTGTAGCTGCGAAGG | 400 | 81 | XM_027958803.3 |

^1^ Primer sequences previously described by Lyahyai and colleagues [18].
