# Supplementary figures and images for: Preclinical serum alterations and tissue changes in protein and gene expression of early cerebrospinal fluid-validated biomarkers in scrapie
Source: Vet Res. 2026 Jun 2;57:98. doi: 10.1186/s13567-026-01759-1 (PMC13231744; doi:10.1186/s13567-026-01759-1)

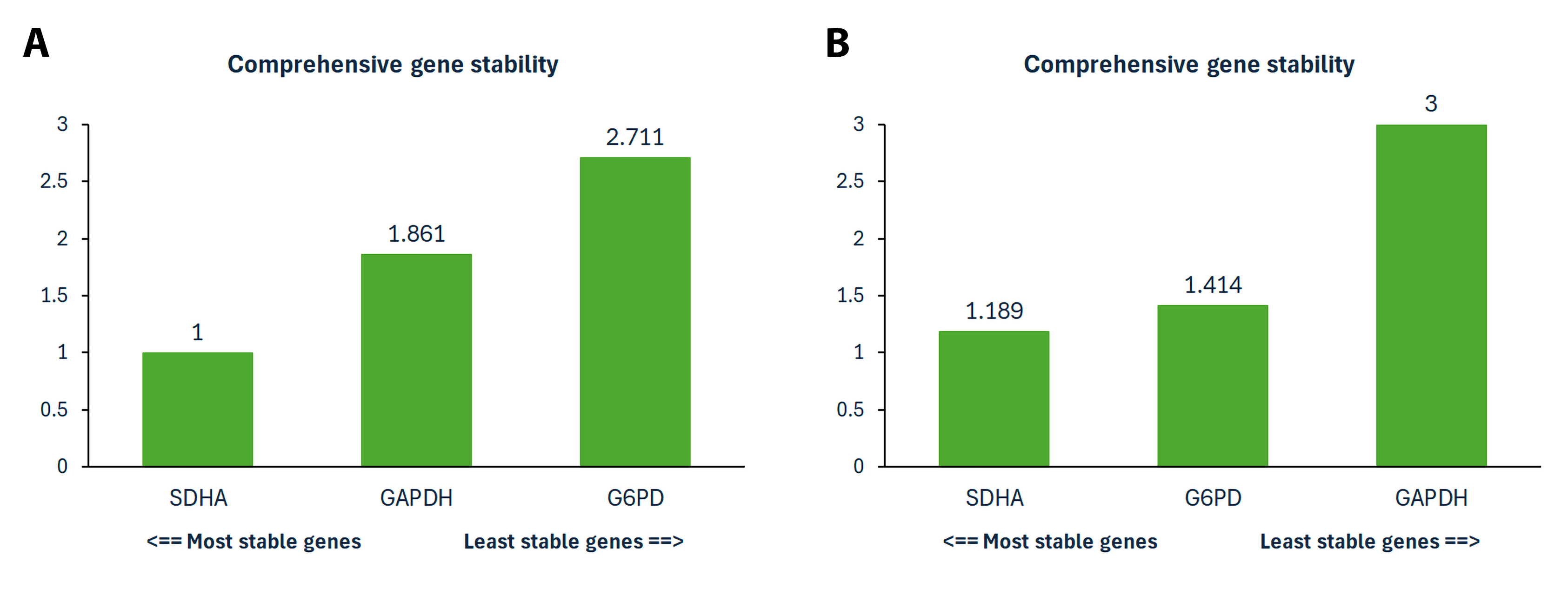

Supplement: Supplementary file 3 — Additional file 3. RefFinder results for the potential housekeeping genes analysed, in obex (A) and thalamus (B). Ranking of the overall stability of the potential housekeeping genes, calculated based on the geometric mean of ranking values, derived from multiple computational algorithms. [file 13567_2026_1759_MOESM3_ESM.tiff]

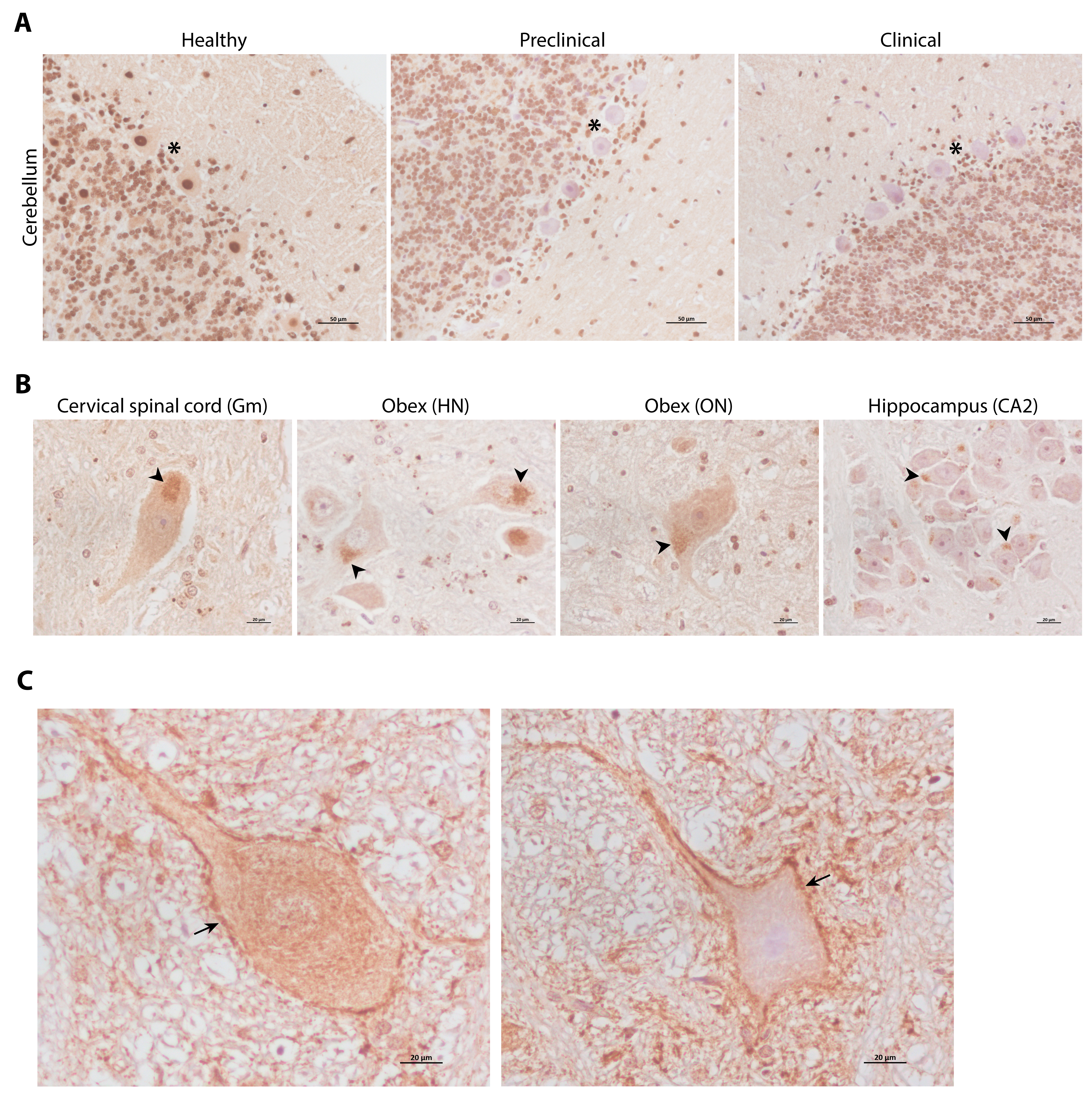

Supplement: Supplementary file 4 — Additional file 4. Distinct immunohistochemical staining patterns of SYNCRIP, CTSD and SPP1 in specific central nervous system regions. A High-magnification immunohistochemical (IHC) images of the cerebellar cortex showing SYNCRIP distribution in Purkinje cells (asterisks) from healthy, preclinical and clinical scrapie-affected sheep. Marked immunoreactivity is observed in healthy Purkinje cells, while a complete loss of staining is evident in scrapie-affected Purkinje cells. B Representative images from healthy animals showing distinct granular cytoplasmic aggregates (arrowheads) of CTSD in several central nervous system regions (left to right): cervical spinal cord grey matter (Gm), hypoglossal nucleus (HN) of the obex, inferior olive nucleus (ON) of the obex, and Cornu Ammonis 2 (CA2) of the hippocampus. C IHC detection of SPP1 in the obex of clinically affected animals, highlighting perineuronal immunoreactivity (arrows) in neurons. [file 13567_2026_1759_MOESM4_ESM.tiff]

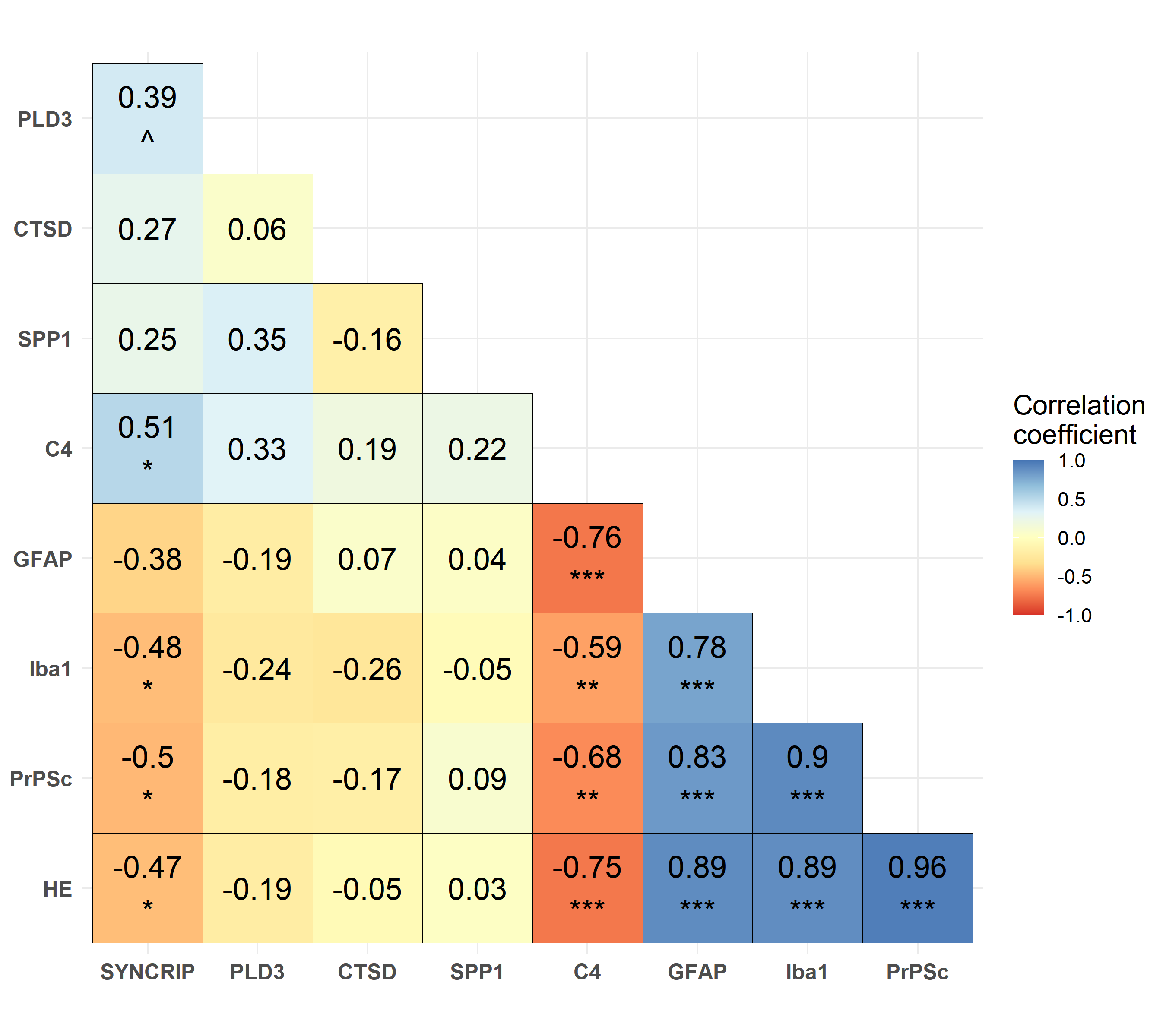

Supplement: Supplementary file 5 — Additional file 5. Correlation matrix between SYNCRIP, PLD3, CTSD, SPP1 and C4 and prion neuropathology, in the cerebellum. Spearman correlation coefficients and significance between the semiquantitative scores of immunohistochemical analysis of the five proteins in healthy (n = 7) and preclinical (n = 5) and clinical (n = 7) naturally scrapie-affected sheep and their neuropathological scores: PrPSc deposits (PrPSc), spongiosis (HE), astrogliosis measured with the glial fibrillary acidic protein (GFAP), and microgliosis measured using the ionised calcium-binding adaptor molecule 1 (Iba1). *p-value < 0.05, **p-value < 0.01, ***p-value < 0.001, ^p-value < 0.1 (tendency). [file 13567_2026_1759_MOESM5_ESM.tiff]
